# Motor imagery classification using sparse representations: an exploratory study

**DOI:** 10.1038/s41598-023-42790-y

**Published:** 2023-09-20

**Authors:** José Antonio Alves de Menezes, Juliana Carneiro Gomes, Vitor de Carvalho Hazin, Júlio César Sousa Dantas, Marcelo Cairrão Araújo Rodrigues, Wellington Pinheiro dos Santos

**Affiliations:** 1 Escola Politécnica da Universidade de Pernambuco, Recife, Brazil; 2 Neurobots Research and Development Ltd, Recife, Brazil; 3 Departamento de Fisiologia e Farmacologia, Universidade Federal de Pernambuco, Recife, Brazil; 4 Departamento de Engenharia Biomédica, Universidade Federal de Pernambuco, Recife, Brazil

**Keywords:** Biomedical engineering, Computer science

## Abstract

The non-stationary nature of the EEG signal poses challenges for the classification of motor imagery. sparse representation classification (SRC) appears as an alternative for classification of untrained conditions and, therefore, useful in motor imagery. Empirical mode decomposition (EMD) deals with signals of this nature and appears at the rear of the classification, supporting the generation of features. In this work we evaluate the combination of these methods in a multiclass classification problem, comparing them with a conventional method in order to determine if their performance is regular. For comparison with SRC we use multilayer perceptron (MLP). We also evaluated a hybrid approach for classification of sparse representations with MLP (RSMLP). For comparison with EMD we used filtering by frequency bands. Feature selection methods were used to select the most significant ones, specifically Random Forest and Particle Swarm Optimization. Finally, we used data augmentation to get a more voluminous base. Regarding the first dataset, we observed that the classifiers that use sparse representation have results equivalent to each other, but they outperform the conventional MLP model. SRC and SRMLP achieve an average accuracy of $75.95\%$ and $82.51\%$ respectively while the MLP is $72.38\%$, representing a gain between $4.93\%$ and $14\%$. The use of EMD in relation to other feature processing techniques is not superior. However, EMD does not influence negatively, there is an opportunity for improvement. Finally, the use of data augmentation proved to be important to obtain relevant results. In the second dataset, we did not observe the same results. Models based on sparse representation (SRC, SRMLP, etc.) have on average a performance close to other conventional models, but without surpassing them. The best sparse models achieve an average accuracy of $95.43\%$ among the subjects in the base, while other model reach $98.33\%$. The improvement of self-adaptive mechanisms that respond efficiently to the user’s context is a good way to achieve improvements in motor imagery applications. However, other scenarios should be investigated, since the advantage of these methods was not proven in all datasets studied. There is still room for improvement, such as optimizing the dictionary of sparse representation in the context of motor imagery. Investing efforts in synthetically increasing the training base has also proved important to reduce the costs of this group of applications.

## Introduction

The brain–computer interface (BCI) has enabled the control or communication with various devices through brain sensors^1^ and opens space for the most varied applications such as touch-free text input systems, controls for: wheelchairs, cursors, prostheses and exoskeletons and virtual reality systems^2^. The EEG plays an important role in several types of brain applications and was popularized for being non-invasive. In addition to its common use in disease diagnosis, sleep assessment and neurofeedback, the EEG has also been widely used in BCI applications^3^, especially using motor imagery (MI-BCI), that consists in limb movement imagination. This cognitive task is reflected in the motor cortex (central surface area of the brain) and occurs in waves of varying frequencies and can represent the imagination of the limbs: right, left hands and feet. Identification and classification can be useful for the creation of interfaces that allow the communication of people with some communication and movement disability.

Because the cortex is the brain region closest to the skull, the EEG becomes a good alternative for identifying motor imagery in a non-invasive way. However, there are challenges, as it is not simple to recognize patterns in the brain signal and classify them as motor imagery. That is why numerous computational methods are researched in order to obtain more accurate results. In this sense, we seek to identify and classify motor imagery in order to have a set of brain commands that will compose the interface. However, the nature of the brain signal picked up by the EEG is non-stationary and poses challenges. The signals are so diverse and time-varying that the usefulness of pattern recognition computational models is limited to the time close to the signal collection. Furthermore, a long recording time is required to achieve good performance for these models. Which in practice can make the user reluctant to use it^4^.

Classifications based on sparse representations of some EEG feature (SRC)^5,6^ are increasingly useful to obtain good performance from untrained patterns^7^. This way, allowing a smaller volume of data to be necessary in the training process of a classification model^2,4,8^ seek to improve this approach by focusing on optimizing and learning dictionaries for sparse representation^9–11^, in turn, choose to focus on improving the features that will be represented.

Empirical mode decomposition (EMD)^12^ is characterized as a good method to contribute to the generation of features extracted from non-stationary signals. Thus, several works use it in the BCI based on Motor Imagery^13–16^. This method presents itself as an option to be combined with SRC and which has not yet been explored.

Taking into account that the motor imagery problem is dependent on each subject, due to neuroplasticity characteristics, requiring customized solutions. Therefore, several works investigate the optimization of windows for each individual, combining this approach with features extracted from common spatial patterns (CSP)^17–26^ Additionally, the frequency separation can also be optimized, aiming to increase the recognition accuracy of imagined movements, also employing features extracted from CSP^10,27–29^. In this exploratory research, we seek to investigate the use of empirical mode decomposition (EMD) and sparse classifiers in the task of pattern recognition in motor imagery. Our hypothesis is that the decomposition of the EEG signal using EMD is more adequate to the non-stationary nature of these signals, and may generate competitive results when appropriate classifiers are used for the sparse representation provided by this approach.

We propose to evaluate the use of SRC of features derived from EMD, in order to verify the hypothesis that this combination is promising in the classification of Motor Imagery in a multiclass problem. We compare the SRC model with a conventional model (MLP). We also evaluated a hybrid approach for classification of sparse representations with MLP (RSMLP). For both approaches we use features derived from EMD. However, we also extract these features directly from the conventional frequency bands, bypassing the application of EMD, in order to arrive at a second comparison. To overcome the difficulty imposed by the base size limitation, we used data augmentation. Feature selection methods were used to select the most significant ones, specifically Random Forest and particle swarm optimization (PSO).

The structure of the subsequent sections is organized as follows: in “Related works” section, we comment on works related to MI-BCI and particularly related to SRC and EMD techniques; in “Materials and methods” section, we present the used materials, methods and theoretical concepts necessary for a good understanding of this work; in “Results” and “Discussion” sections we show the results and discussion. Finally, our conclusions and future works are described in “Conclusion” section.

## Related works

### Motor imagery based BCI

Several works have proposed methods aiming to improve motor imagery classification. ^30^experimented different techniques to adjust CNNs, for instance. In this study, they tested a subject-independent categorization to overcome the small amount of EEG signals per participant. The main idea of the work is to train the network with data from different subjects. Then, using the data from the chosen subject, the pre-trained model is altered and adjusted. With that in mind, the researchers tested a variety of network adaptation mechanisms: classifier optimization, adaptation of convolutional layers, and multiple proportions of training (the number of layers retrained with the data of the patient). In order to test the proposed method, they used EEG signals from 54 healthy participants who imagined right and left-hand movements. Spatial and temporal filters with max-pooling, three convolutional blocks, and a fully connected layer with Softmax were employed in the CNN. Finally, the study found that, when compared to standard methods (with subject-specific training), the proposed method had a $32.50\%$ greater accuracy. In addition, the best results were achieved with adaptation of three of the four convolutional layers, as well as the Softmax layer. This means that only the first layer was properly trained with data from other patients.

As in the previous work, ^31^also proposed a subject-independent training approach. The authors idea was to train BCIs with data from numerous participants and transfer the learning to classify a new subject. Thus, the authors modified the classic CSP method and applied a bandpass filter. With this, they designed a structure with greater discriminative ability, called separate channel convolutional network (SCCN). The methodology was tested on dataset 2b from the BCI Competition IV (2008). Two measures were used to evaluate the results: accuracy and Information Transfer Rate (ITR). The proposed technique had an ITR of 0.83 and an average accuracy of $64\%$. Unfortunately, simpler classifiers, such as LDA, produced better results ($65\%$).

^32^tested another novel technique: the combination of numerous CNN models with varying depths and filters to improve motor imagery categorization results. In this case, the CNN models were based on the AlexNet architecture. The features extracted by the various models are then combined and sent into an MLP (MCNN Method) or an autoencoder as an input (CCNN Method). The authors experimented four types of CNNs, ranging from one to four convolution blocks and max-pooling, as well as filters with sizes ranging from 10 to 30. The CNN models were trained and tested with two datasets: the High Gamma Dataset, which is made up of 20 volunteers’ EEG signals, and the BCI IV-2a (BCID) dataset. The BCID is made up of electroencephalogram (EEG) data from nine participants who imagined four different sorts of movements: right hand, left hand, foot, and tongue. In terms of training, the study looked at two approaches: subject-specific training and training based on signals from all volunteers. Therefore, based on the BCID, the first approach had an average accuracy of $75.7\%$. In the second approach, the CCNN outperformed the others, with an average accuracy of $55.34\%$.

In turn, ^33^concentrated on two methods: modifying the CNN’s kernel dimensions, and data augmentation for each subject. The researchers looked at how kernel size differed between participants and between imaging sessions for the same subject. This analysis found a $10\%$ difference in accuracy with kernel variations. Based on these findings, they presented a CNN with a hybrid convolution scale, named HS-CNN. Furthermore, the paper also presented and tested a new way of data augmentation, in which signal windows are recombined in the time and frequency domains. The researchers used two BCI Competition IV databases to validate the method: 2a and 2b. Each dataset is made up of nine healthy people who imagined four and two types of movements, respectively. The proposed method has an average classification accuracy of $87.6\%$, with improvements of up to $23.25\%$ for dataset 2a and $19.7\%$ for dataset 2b.

In contrast, ^34^invested in new methods of representing EEG signals for classification of motor imagery. Instead of using EEG signals in one dimension, the authors proposed a way to represent them in three dimensions. In this way of representing, the authors sought to keep the spatial distribution information of the electrodes in addition to the temporal information. For feature extraction and classification steps, they developed a CNN also in 3D (Multi-branch 3D CNN). The idea with this architecture is to combine multiple CNNs with different receptive fields. Finally, this methodology was tested with data from the BCI Competition IV 2a database, and the Multi-branch 3D CNN achieved superior results were compared with a Small, Medium and Large Receptive Field networks only.

### EMD and SRC MI-BCI

Recognizing patterns from sparse representations, although it emerged in the field of image classification^35,36^, is becoming increasingly useful in the classification of biological signals. ^37^review methods of sparse representations of EEG signals for detecting epilepsy. ^7^propose a classification model based on the smallest residual produced in the reconstruction of a sparsely represented sample. The technique is combined with extreme learning machine (ELM), a random-weighted one-layer neural network^38–40^, to classify myoelectric signals in the control of prostheses. Signals were collected from two amputees, with and without a prosthesis, in addition to eight healthy individuals. The effectiveness of the method was evaluated both in offline and online mode. In the first, the subjects perform suggested movements in random positions of their amputated arm, varying the positions in the three dimensions. The classifier is trained however using only one position while the others are used as a test. When the ELM does not sufficiently discriminate an SRC move is used to resolve the impasse between the doubtful classes. In online mode, subjects perform suggested moves in four untrained positions while receiving real-time feedback using the same classifier combination as in the previous mode. The method was compared with six other classifiers trained with three feature groups. In all groups of subjects, the proposed method was the one that obtained the lowest error rate with significant performance improvement ($p < 0.001$) for the untrained positions.

This SRC model based on the smallest residual is still little applied in MI-BCI studies. Therefore, it is of interest to this work to evaluate the technique in this new context. The sparse sample is used to reconstruct the original sample which will be classified according to the class that produced the smallest residue during its reconstruction. A better description of the method can be found in the "Feature selection" section.

^5,6^appear among the first studies exploring SRC in the classification of motor imagery. Since then, sparse representation has been increasingly used in this type of problem. For us, approaches that seek to improve the features that will be sparsely represented are interesting to situate the state of the art. However, these approaches do not classify the samples themselves, but their sparse representations. This differs from the classification by the smallest^7^ residue studied in our work. But we find it appropriate to cite the pioneering studies on the combination of sparse representation techniques in the context of MI-BCI.

^9^propose a classification mechanism for the sparse representations of optimized features in space, time and frequency. The best EEG channels are selected based on relative entropy (spatial optimization), the signals from this subset of channels are decomposed into several overlapping frequency bands (frequency optimization) and time-segmented into overlapping windows (temporal optimization). Features based on the CSP are then extracted and the most significant are selected by sparse regression. Finally, a dictionary is generated and optimized to aid in sorting. The approach was evaluated in two databases and obtained an improvement of 21.57% and 14.38% compared to other methods applied in the same databases. In addition to comparison with other methods, the study assesses the impact of dictionary optimization by comparing SRC results with and without dictionary optimization, obtaining a significant improvement of 9.44%.

^10^use the same time-frequency feature optimization method as the previous study^9^ but uses sparse representation as an feature selector technique to train an SVM classifier^41,42^. The approach was evaluated in three databases where mean accuracies of 88.5%, 83.3% and 84.3% were obtained, obtaining significant improvement ($p<$ 0.01) in relation to the other compared methods.

EMD is another technique that can be used to improve features for classifying motor imagery, but it has not yet been combined with the SRC technique ^43^use EMD to decompose the signal into five components, called Intrinsic Mode Functions (IMF), to extract three features. The resulting feature vector is used to train an artificial neural network and obtains good results in discriminating four classes of motor imagery. The technique is compared to Discrete Wavelet Transform (DWT). EMD traits achieve an average accuracy of 90.02%, while DWT traits average an accuracy of 84.77%. ^44^have a similar application, but extracts up to seven features from the produced IMFs. The proposed model consists of a three-layer hierarchical SVM classifier, which surpasses the compared model (traditional SVM). ^12^offer an analysis of the applicability of EMD in the identification of motor imagery. In addition to generating features, the technique can also be used to remove artifacts^45^. ^46^use EMD to compose an enhanced EEG signal, considering the average frequency of the MFIs. Three Hjorn parameters plus the absolute power are extracted from the new signal to compose the feature vector. The LDA classifier is then applied in the classification. It was observed that the technique can improve on average $10.5\%$ of classification accuracy. ^47^evaluate EMD multivariate extension (MEMD) in MI-BCI. Searches for peaks and troughs in n directions (multichannels) of the input signal improving the location of frequency information. SVM is applied in classification.

Regarding EEG signals classification, successful approaches have been adopting sparse representation, since these applications are strongly dependent on frequency representation, which tends to present sparse features obtained by sequential filtering as the most appropriate candidates to represent this information. For instance, ^48^proposed a method to detect seizure EEG signals by empirical mode decomposition (EMD) and sparse representation. The EMD method is used to process EEG signals for generating IMFs. Then frequency features of IMFs are extracted as the dictionary in sparse representation based classification (SRC) scheme to realize reduction of the data dimension and calculation cost. The generated dictionary is automatically optimized by a clustering algorithm, similarly to^17,18^, that adopted common spatial patterns (CSP) instead of IMFs. Then, ^48^demonstrated that this approach is able to detect the seizure EEG signals with a considerably high accuracy: up to 99%. According to^48^, this approach is fast and practical for the treatment of epilepsy in practice. We understand that combining the two approaches EMD and SRC can help in the classification of motor imagery.

An SRC classifier depends on its dictionary matrix, and its construction is an important step for good performance. ^5^uses features based on CSP to demonstrate that the property of incoherence between signals collaborates so that a new sample can be predominantly represented by one of these signals. Examining the EMD, it is important to note that the IMFs tend to have different frequencies, which guarantees a degree of incoherence between them most of the time, since the coherence is often calculated in terms of frequency, comparing the spectral components in each frequency. Therefore, signals with different frequencies have different spectral characteristics and do not share similar information at these frequencies. However, it is a particularly difficult point to guarantee a high level of incoherence at all times. The IMF components can have a wide frequency range in some cases, due to the non-stationary nature of the signals, being impossible to determine in advance due to the empirical nature of the method. However, by observing the components, we can see that most of the time these frequencies are different. The interest in associating EMD and SRC in this work is due to the particular advantages of combining EMD with brain signals, due to its non-stationary character. Also for the important results achieved with sparse representations in the classification of these signs pointed out by many studies. Therefore, it is in our interest to explore this combination that is still little evaluated and to document our findings in this study. However, it is worth remembering that this approach has already been evaluated in the context of signs in epilepsy by^48^, confirming the feasibility of the technique.

## Materials and methods

### Databases

**Figure 1:**
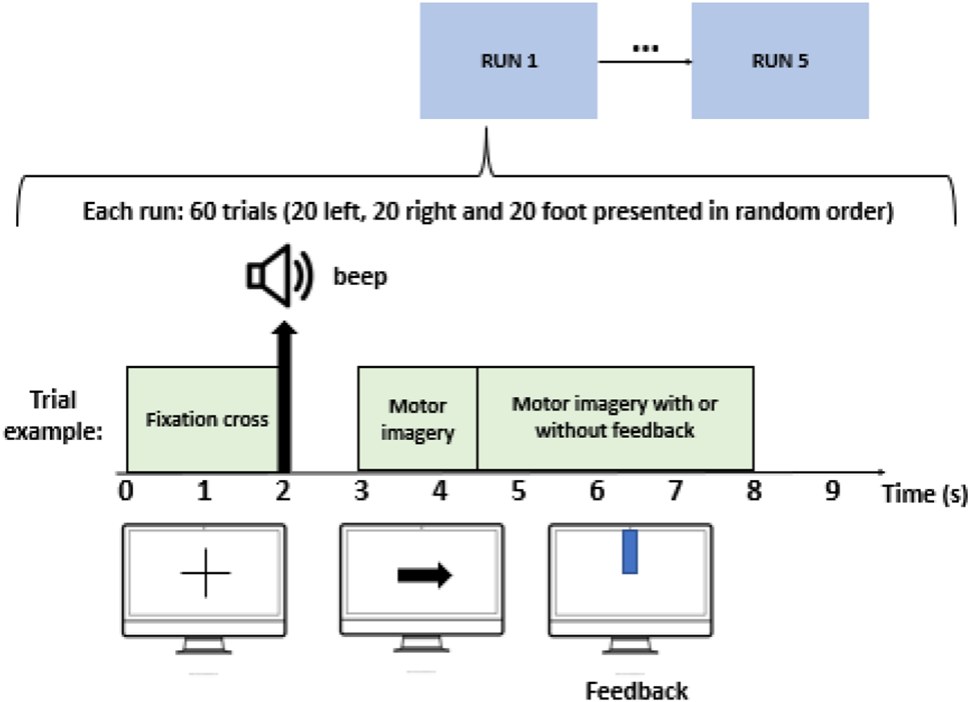
Database acquisition protocol: The collections were performed in 5 sessions, on alternate days. Each of the sessions is composed of 60 trials of 8  s, with 20 trials referring to the right hand, 20 to the left hand and 20 to the foot. At the beginning of each trial, a fixation cross appeared on the screen, followed by a beep, both indicating an impending motor imagery instruction. Then an arrow appeared on the screen, indicating which class should be imagined. Finally, the acquisition of imagination was done both with and without visual feedback.

Two datasets were used in this work. First database was provided by the Neurodynamics Research Group of the Federal University of Pernambuco. It consists of 21 EEG signal collections from a single healthy adult male. The collections were performed on alternate days and each one consists of 60 trials of 8 s. In each trial, in turn, the motor imagery of the right, left or feet is performed. EEG data were collected through the g.Hamp amplifier set to a sampling rate of 256Hz. In addition, 27 active channels were distributed over the premotor, motor and sensoriomotor cortex. After preparation and placement of the electrodes, the data acquisition protocol was performed as follows (Fig. 1):

1. The individual wearing an EEG sits in front of a Monitor.
2. In a few moments, a cross appears to alert you of an impending instruction.
3. An arrow will randomly indicate which activity to do in that trial. E.g.: arrow to the right will indicate motor imagery of the right hand, etc.
4. The individual performs the indicated instruction within 8 s of the trial. It may or may not receive real-time feedback on the quality of its execution.
5. The screen is completely white and the subject rests for 4 s.
6. The process is repeated until completing 60 trials.

The study was conducted in compliance with the guidelines of Resolution 466/2012 of the National Health Council and the Declaration of Helsinki of 1964 and approved by the Ethics Committee of the Health Sciences Center of Federal University of Pernambuco, CAAE number 79271517.2.0000.5208, Recife, Brazil. All participants signed the Informed Consent Term (ICT) prior to the start of the research. Patients were informed of the goals and procedures, as well as the risks and benefits, and could interrupt the assessments at any time, without the need for further explanation.

The second base used was the BCI Competition IV dataset 2b, which is widely used in the classification of motor imagery of the upper limbs. Dataset 2b provides signals from 3 brain sites: C3, Cz and C4, organized into several trials labeled with the imagery class. And it consists of 5 sessions of 9 subjects. Each session has several attempts for each motor imagery class that last approximately 8 s. More details about this base can be found in^49^. In this work we used the 5 sessions of each subject. For each trial we used the first 3 s of imagery.

### Proposed classification methodology of EEG signals by sparse classifiers and empirical mode decomposition

**Figure 2:**
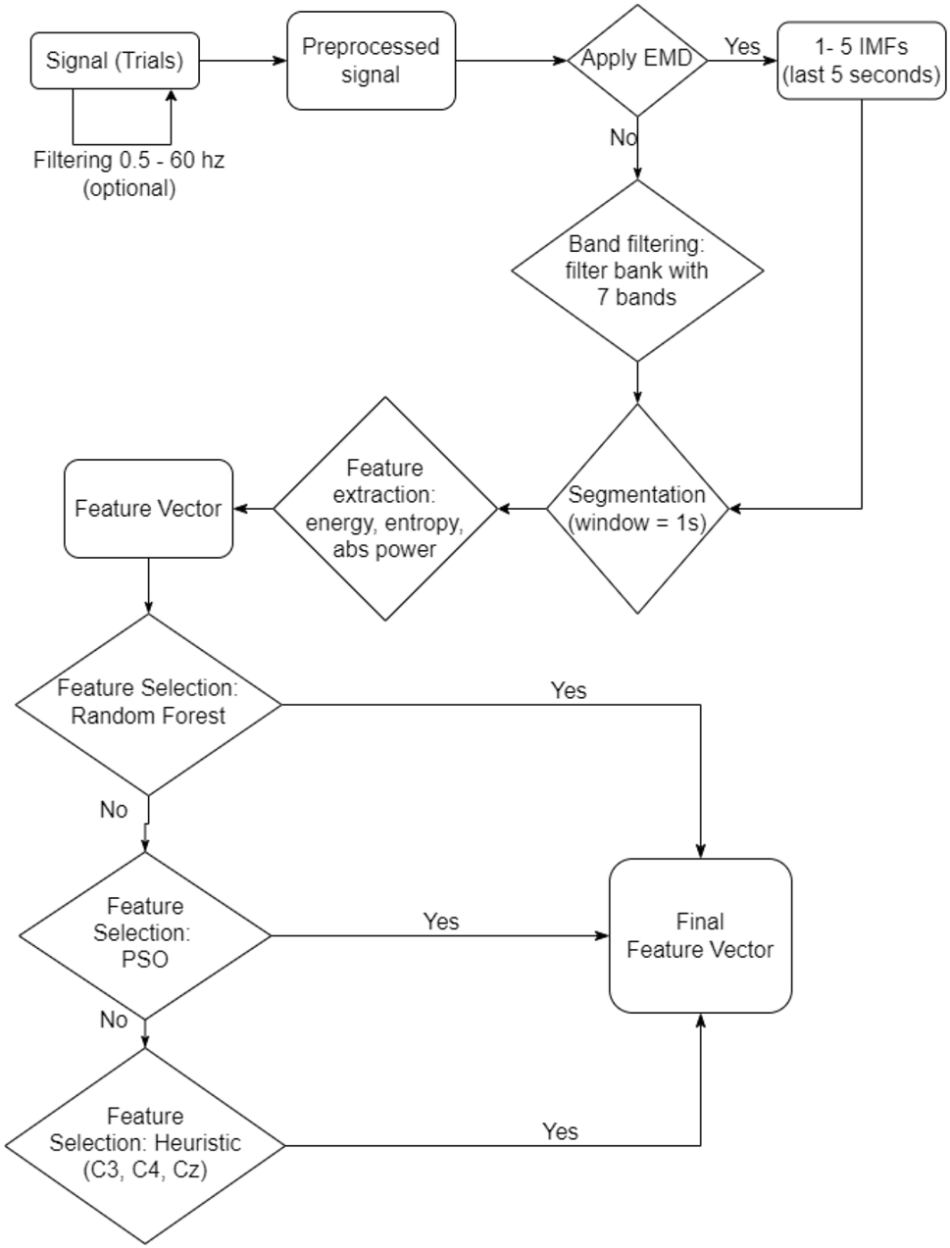
Trial processing step flow, 8-s window of EEG signal (“Databases” section). The flow presents the processing possibilities to obtain the feature vector used in this work. First, the signal is normalized, then you choose to decompose the signal by EMD (setups 1–5, Table 1) or into frequency bands (setup 6, Table 1) which will generate signal components that will be segmented into 1 s windows. For each segment 3 features are extracted (energy, entropy and absolute power). In dataset 1, the feature vector will have 2025 features in total (27 $\times$ 5 $\times$ 5 $\times$ 1) when using EMD and 5103 features (27 $\times$ 7 $\times$ 9 $\times$ 3) when use optimization time-frequency. In dataset 2, the feature vector will have 135 (3 $\times$ 5 $\times$ 3 $\times$ 3) when use EMD and 441 (3 $\times$ 7 $\times$ 7 $\times$ 3) when use optimization time-frequency. This requires the selection of these features, which can be done in three ways: using Random Forest, PSO or manual. The resulting vector will serve as input for training and validating the classifiers.

In this work, we propose to evaluate the effectiveness of sparse representations in classifying motor imagery from features based on EMD. For this, we use processing, extraction and selection methods of features already related to the manipulation of EEG signals. Figure 2 shows how these methods are embedded in our goal flow. The “Signal pre-processing and feature extraction” section brings them up in detail.

### Signal pre-processing and feature extraction

The first step of our processing consisted of separating and organizing the trials for each collection. At this stage the signal could be filtered at 0.5 and 60 Hz and normalized. EMD (Empirical Mode Decomposition) was applied to all channels. At least 5 IMFs (Intrinsic Mode Functions) were obtained, they approximate the conventional frequency bands (delta, theta, alpha, beta and gamma) but are more suited to the non-stationary nature of the EEG signal. However, it was also an option to use a filter bank with the following bands: 4–8 Hz, 8–12 Hz, 12–16 Hz, 16–20 Hz, 20–24 Hz, 24–28 Hz, 28–32Hz . These seven bands may allow for better adaptation of a subject’s signal processing. Especially the 4–12 Hz and 12–16 Hz bands, which are conventionally called alpha and slow beta, are related to motor activity. Abordagens semelhantes podem ser encontradas em^10,27–29^.

Once the trial was decomposed or filtered, we segmented it into 1 s windows and used only the last 5 s to extract features. We can consider that the initial seconds of a trial contain still scattered imagery information, since the individual was waiting for instructions, so we chose to discard the first 3 s of the trial. The extracted features were energy, sample entropy and absolute power of the segments.

#### Empirical mode decomposition—EMD

EMD is a self-adaptive method that decomposes the signal without leaving the time domain. It is useful for analyzing non-stationary and non-linear signals such as brain signals. It consists of breaking the signal into a finite number of intrinsically-mode functions (IMF)^12^. For a given signal *x*(*t*) it can be decomposed into *n* IMF *c*(*t*) and a residue *r*(*t*).

1 $$\begin{aligned} x(t) = \sum _{i=1}^{n} c_i(t) + r(t) \end{aligned}$$

IMF are in this case adaptedly derived directly from the input signal. An IMF must satisfy the following requirements:

1. The sum of the number of maximums and minimums of the IMF must be equal to the number of zero crossings or differ at most by one;
2. The mean between the envelope defined by the local maximum and the envelope defined by the local minimum must be zero at any point in the IMF.

**Figure 3:**
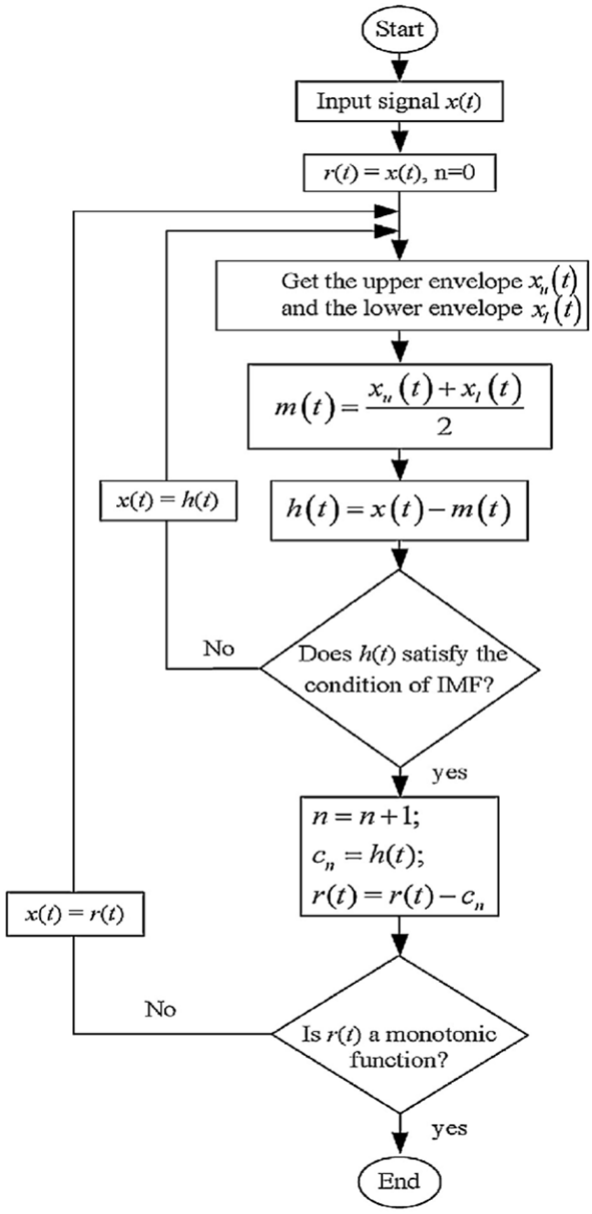
Flowchart of empirical mode decomposition algorithm^43^.

**Figure 4:**
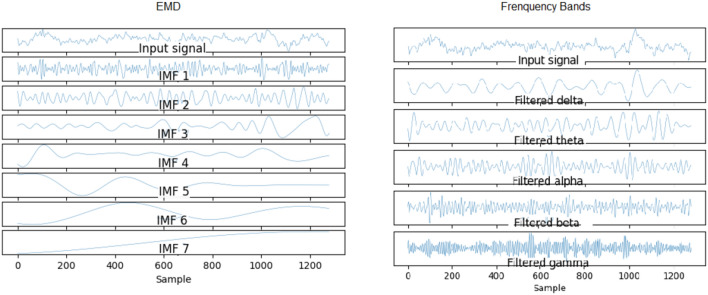
Example of empirical mode decomposition that generated 7 empirical functions: 6 IMF and 1 residual.

^43^presents a good scheme of how the algorithm to obtain the IMFs works and can be seen in Fig. 3. At first, the search for an IMF is performed on the input signal. This search consists of determining local maxima and minima forming an empirical function. When this function meets the criteria described above, it is considered an IMF. From there it is subtracted from the original signal and the process is repeated on the resulting signal. The process ends when the resulting signal is a monotonic function. As it is empirical in nature, the amount of IMFs produced by a signal is not delimited and may vary between different signals. However, it is noticed that the first IMFs have a higher frequency while the last ones make up the linear trend of the signal, as shown in the Fig. 4.

### Feature selection

In dataset 1, each trial processed carries information from 27 channels, decomposed into 5 IMFs, segmented into 5 windows of one second, where 3 functions were calculated for each segment. So our feature vector has a size of 27 $\times$ 5 $\times$ 5 $\times$ 3 = 2025 features. In dataset 2, trials has 3 channels, 5 IMFs, 3 windows of one second and 3 functions (3 $\times$ 5 $\times$ 3 $\times$ 3 = 135 features). It is therefore a high number, capable of dispersing the low amount of trials available and making the sorting activity very costly. Then we reduce the vector size using a heuristic approach. It is known by Neuroengineering that certain brain sites are more likely to concentrate motor imagery information. These sites are called C3, C4 and Cz. Both are located close to the central sulcus, in the primary motor cortex and are related to motor activity in the right hand, left hand and feet, respectively. It should be noted that these are the classes we are trying to identify. It was also possible to bet which IMFs would be more likely to contain motor imagery. We chose the 3rd and 4th IMFs because of the similarity^43,44^ with beta and alpha bands, commonly related to motor activity.

So our new vector has 3 $\times$ 2 $\times$ 5 $\times$ 3 = 90 features. We thus mitigate the risk of underfitting signaled by the size of the initial vector and reduce classification costs. However, it was still of our interest to use computational methods of feature selection, motivated by the notion that we could be missing some important information for the discrimination of the three classes of imagery. Added to the complexity of the brain signal, the interconnection of brain areas does not exhaust the possibility of the information we seek to be reflected in other brain sites. Two alternatives to heuristic feature selection are Particle Swarm Optimization (PSO) and Random Forest aided feature selection. These methods are explained below.

#### Particle swarm optimization—PSO

Particle swarm optimization (PSO) is a widely established method that works as decision trees, where features are ranked according to accuracy. The PSO uses an objective function that seeks to minimally penalize ranking performance, keeping it as high as possible with a reduced number of features^50–58^.

The PSO algorithm was inspired by a study of groups of birds looking for a place to build their nests, as well as looking for food. In this case, the movement of the entire pack is based on the overall intelligence of the group. With that in mind, the algorithm initially uses a population of randomly generated particles or individuals, each individual being a candidate for the solution of the fitness function. Each individual is associated with a position vector and a velocity vector. The flock’s trajectory is then guided by two sources of information: the position of the best particle in relation to the aptitude function and knowledge of the places previously visited by each particle. In this way, at each iteration of the algorithm, the positions and velocities of the particles are adjusted towards the best global and individual position^59–63^.

Feature selection by PSO was implemented in the database using 30 particles and k-NN as objective function estimator. The individual particle (parameter $$c_{1})$$ and global consciousness were adopted as 0.5. The inertia factor *w* was 0.9 and the number of neighbors was 30.

#### Random forest for feature selection

In addition to PSO, we test feature selection with decision trees, which can be used in classification and regression problems. In particular, Random Forests are a combination of trees that are built with different samples from the original database. Essentially, each tree is made up of four types of nodes: root, leaf, parent and child nodes. The starting point is the root and the terminal nodes are the leaves. Thus, using such trees, the algorithm makes a decision after following a hierarchical path that starts at the root node and reaches the leaf node. Thus, after the formation of the forest, a new object that needs to be classified is placed for each of the trees to classify. At the end, each tree casts a vote that indicates its decision on the object’s class. The forest then chooses the most voted class for that^64^ object.

In the case of feature selection, the measure of importance of each feature can be calculated according to the reduction of the model’s accuracy when the feature in question is not included in the tree. In other words: decision trees based on less relevant features will have a lower ranking performance^65^. In our study, Random Forests were implemented using 100 trees or iterations.

### Classification

The classification activity consisted of estimating which of the motor imagery classes (right hand, left hand and foot) a trial belongs to. It is an offline classification of trials (8-s activities of intense motor imagination). However, it was also part of this study to explore the classification of segments (1 s in length). This being a simulation of what would be an online application to identify motor imagery.

**Figure 5:**
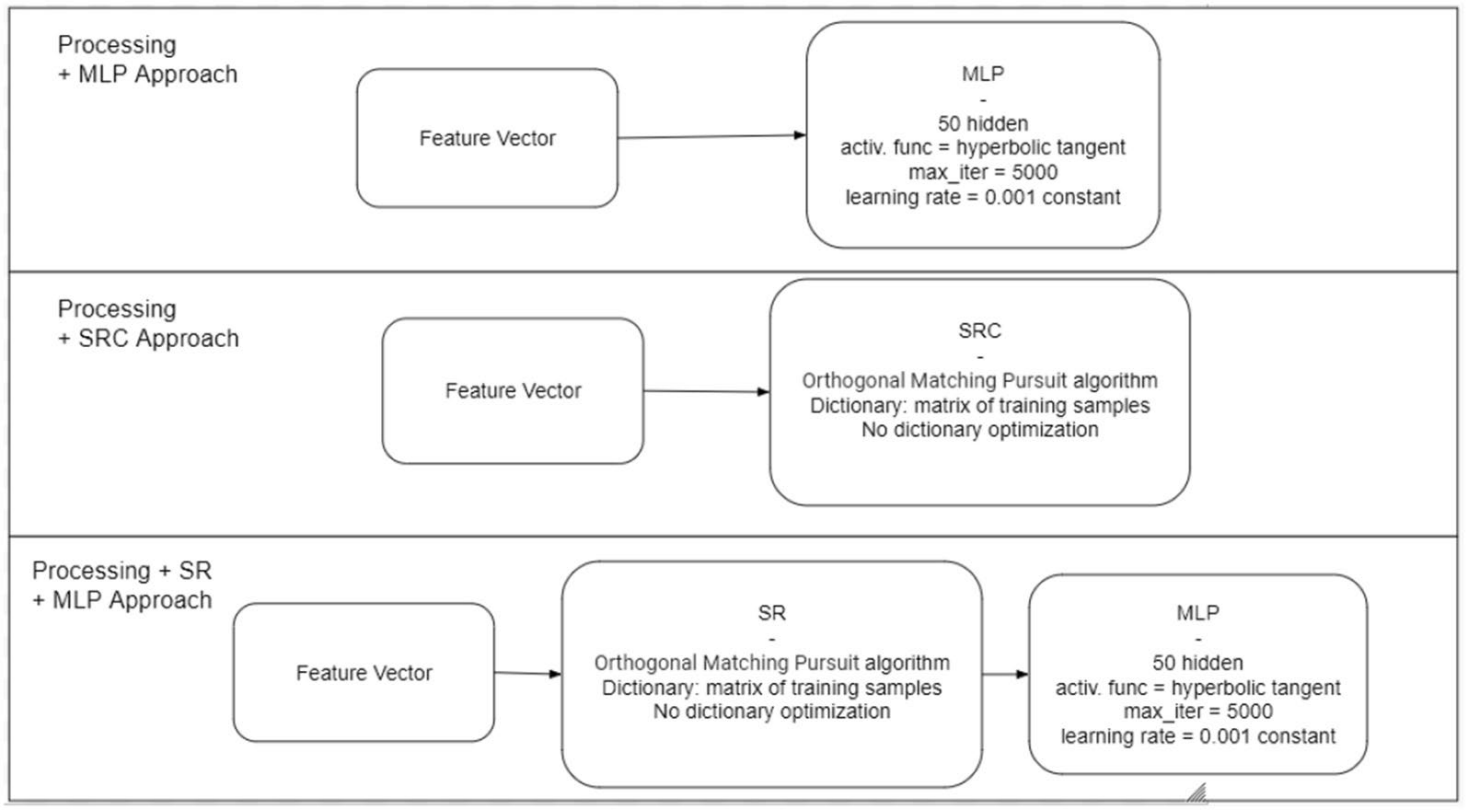
Classification: three approaches for classifying motor imagery were tested, with a view to future comparison. All three approaches went through the feature selection step, differing in the type of classifier used. The first approach consisted of classification with MLP; the second approach used the SRC; the third approach was hybrid, encompassing sparse representation and MLP as a classifier.

Figure 5 shows the different approaches to classification used in this study. Assuming that the SRC holds promise for classifying untrained data, it was necessary to compare it with another “common” classifier. We therefore chose the multilayer perceptron neural network (MLP). However, it was also possible to explore the potential of sparse representations in a classifier other than SRC. We then added to our analysis a hybrid approach that used sparse representation but used MLP for classification.

#### Sparse representation classification

**Figure 6:**
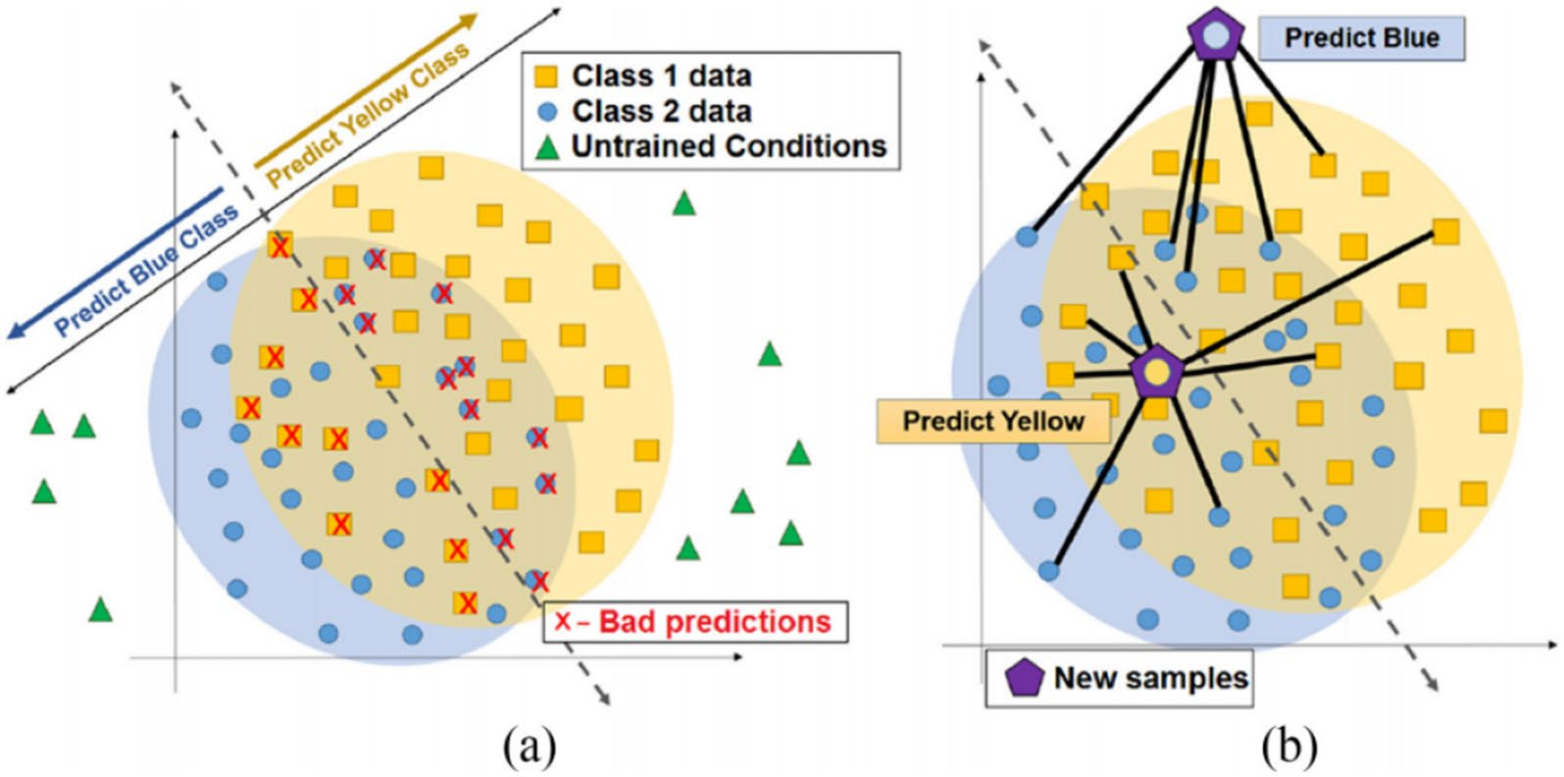
Sparse representation classification scheme. (**a**) Untrained conditions often appear in real situations and do not follow the patterns of the training set. Therefore, boundary classifiers cannot discriminate between them. (**b**) In SRC these data are reconstructed from a minimal subset of training samples and the reconstruction is then classified instead of the original sample. Therefore, all samples from the training base can influence the sparse representation of the untrained sample. This gives you more flexibility in classifying both data within the class boundaries and outside them^7^.

Frequently, the use of classifiers in real situations exposes the model to untrained conditions. The SRC is an algorithm that seeks greater robustness in predicting these untrained conditions. Figure 6 illustrates the idea behind using a sparse representation for classification. While bounds-based algorithms cannot separate data from untrained conditions, in SRC these data are reconstructed from a minimal subset of training samples and the reconstruction is then classified instead of the original sample. An SRC classifies samples based on the residual resulting from the reconstruction of that same sample after it has been sparsely represented. The reconstruction with the smallest residue should determine which class the sample belongs to.

A sparse representation is a linear combination of a set of vectors, called a dictionary, to obtain a sparse vector, which has a large number of null or near-null values^17,18^ The dictionary, in turn, is composed of samples from the training base. These samples already have their features extracted. The classification of a new sample is done by observing the weights of the linear combination that most contribute to the construction of the sparse vector. Members of the correct class are expected to contribute more weight.

Let *D* be a training dictionary, an array composed of $D_i$ partitions, with $i=1, \dots , n$, where *n* is the number of classes, and $D_i$ is composed of the class’s training samples *i*. Thus *D* forms a matrix partitioned by elements of each training class. For example, in a problem to distinguish upper limb motor imagery, the training dictionary can be defined as the union of 2 sets of left and right hand samples: D = [ $D_{left} \mid D_{right}$ ]. A new sample *y* is reconstructed from a linear combination *Dx* where $x = [x_1, x_2, \dots , x_L]^T$ are the weights associated with each training sample. A sparsity constraint will limit the linear combination to a minimal subset of training samples, i.e. most of the weights should be zero resulting in a sparse vector. This restriction is imposed through the optimization problem

2 $$\begin{aligned} x = \mathop {\textrm{argmin}}\limits _x \left\{ \frac{1}{2} ||Dx - y||_2^2 + \lambda ||x||_1\right\} \end{aligned}$$

where $\lambda > 0$ is a sparsity smoothing parameter. Since the transformation ($Dx = y$) will hardly be exact, the residual is then calculated for each class *i*

3 $$\begin{aligned} r_i = ||D_ix_i - y||_2 \end{aligned}$$

and the one with the smallest residual is the predicted class for the *y* sample. Following the example of the motor imagery classification of upper limbs, a new sample (*y*) is sparsely represented ($y_2$) and then $r_{left}$ (by $D_{left} x_{left} - y_2$) and $r_{right}$ (by $D_{right} x_{right} - y_2$), the smallest value between $r_{left}$ and $r_{right}$ will point to the partition ($D_i$) of the dictionary that most contributed to the sparse representation of the sample and therefore the probable class of the new sample.

#### Multilayer perceptron

In 1958, Frank Rosenblatt proposed the perceptron model as the simplest form of an artificial neural network for binary classification. The perceptron consists of a single neuron with synaptic weights and adjustable bias, capable of classifying linearly separable problems^66^. Multilayer Perceptron (MLP) is a generalization of the Rosenblatt perceptron and consists of several layers: input layer, one or more hidden layers, and an output layer. Having additional hidden layers allows MLPs to solve more complex problems^66,67^.

The training of an MLP is carried out in a supervised manner and aims to adjust the synaptic weights, so that the output of the network approaches what is expected. To carry out this adjustment, the most used method is the error retropropagation algorithm, consisting of two phases. In the propagation phase, an output is obtained from an input. Then, in the backpropagation phase, the error is calculated using the obtained and desired outputs. Through the error obtained, it is possible to adjust the weights and iteratively minimize the^68^ error. MLPs have been commonly used in medical applications, such as in the diagnosis of cancer^69–75^, diabetes^76^, multiple sclerosis^77^, and in the monitoring and diagnosis of Covid-19^78–80^.

### Experiments settings

The scheme shown in Fig. 2 enabled a series of options for signal processing that served as a parallel input for the classifiers shown in Fig. 5. We started with a heuristic approach first and then made variations, combining other processing options as they proved promising. Table 1 lists the combinations chosen in this work. The 10-fold cross-validation was performed three times for each configuration evaluated, totaling 30 scores per setup.

**Table 1:** Groups of performed experiments.

Experiment ID	Sample type	Channels	IMF	Feature selection
Setup1	Trial	C3, C4, Cz	3,4	None
Setup2	Trial	All	1,2,3,4,5	PSO
Setup3	Trial	All	1,2,3,4,5	Random forest
Setup4	Segment	All	1,2,3,4,5	Random forest
Setup5	Resampled trial	All	1,2,3,4,5	Random forest
Setup6	Trial	All	None (frequency bands)	Random forest

Considering the type of sample, the trial is the default sample for most setups (setup 5 classifies the trial but using a resampler mechanism to increase the base). In setup 4 the classified sample is the 1 s segment of the signal, instead of the trial. The segment does not compose the feature vector, as in the other configurations, therefore reducing the vector size (for exemple, dataset 1 reduce to 405 features = 27 channels $\times$ 5 components $\times$ 3 features), but increasing the base sample volume. Considering the type of decomposition, setups 1–5 apply EMD and select up to five IMFs to compose the feature vector.

Setup 6 is the only one that doesn’t use EMD. It uses optimized time-frequency to determine which set of filters and windows generate the best performance for a given subject^10,27–29^. In this approach, we use a filter bank with 7 bands of 4 Hz length each (4–8 Hz, 8–12 Hz, 12–16 Hz, 16–20 Hz, 20–24 Hz, 24–28 Hz, 28–32 Hz). We segmented the signal into 1-s windows with 50% overlap in the useful seconds of each trial. For dataset 1 this means that there are 5103 features in all (27 channels $\times$ 7 bands $\times$ 5 s separated into 9 segments $\times$ 3 functions). For dataset 2 there will be 441 features (3 channels $\times$ 7 bands $\times$ 4 s separated into 7 segments $\times$ 3 functions). Due to the high volume of features it is very important to use an efficient feature selection method, we use the selection based on RandomForest parameterized with 100 trees.

In terms of channel and feature selection, setup 1 is the heuristic approach described in “Feature selection” section. It is the approach with the smallest feature vector (= 90) and therefore the least expensive. The remaining setups apply computational mechanism for feature selection. The PSO used in setup2 was configured for 30 particles, the k-NN (k = 4) served as an estimator for the objective function and we assigned the following values to the parameters: c$_1$ = 0.5, c$_2$ = 0.5, w = 0.9, k = 30, *p* = 2. Random Forests in turn were used with 100 trees. We can note that Random Forest was the most frequent method of feature selection used. This is due to its speed compared to PSO and therefore it is less expensive.

The feature vector produced by each setup was then evaluated in 3 classifiers: MLP, SRC and SRMLP (Fig. 2). MLP was trained with 50 neurons in the hidden layer, while OMP was the algorithm used in approaches that included sparse representation of the feature vector.

### Metrics

We consider two metrics to evaluate the performance of the experiments: the accuracy and the Kappa index. Accuracy is the ratio between the number of correct predictions and the total number of classified samples. In other words, it is the probability of the classifier correctly indicating the class imagined by the patient or user. Its mean and standard deviation were useful in analyzing groups of experiments. The accuracy was calculated using the Eq. 4 below:

4 $$\begin{aligned} Accuracy = \frac{TP + TN}{TP + TN + FP + FN} \end{aligned}$$

where TP indicates true positives, TN indicates true negatives, FP indicates false positives and FN indicates false negatives.

The Kappa index, in turn, is a metric capable of dealing with multi-class problems, such as the one proposed here. It assesses the level of agreement between data sets, with the maximum value being 1, where values above 0.75 suggest excellent agreement and values between 0.40 and 0.75 a median agreement^81,82^. The Kappa index can be calculated using the Equation 5 below:

5 $$\begin{aligned} k = \frac{\rho _o - \rho _e}{1 - \rho _e}, \end{aligned}$$

where $\rho _o$ is the observed agreement, or accuracy, $\rho _e$ is the expected agreement, defined in the Equation 6.

6 $$\begin{aligned} \rho _e = \frac{(TP + FP)(TP + FN)+(FN + TN)(FP + TN)}{(TP + FP + FN + TN)^2}. \end{aligned}$$

## Results

### Database 1

Table 2 summarizes the value of the metrics studied for each setup presented in the “Experiments settings” section. In setup2 that uses PSO Search, 1250 of 2025 features were selected (27 channels $\times$ 5 IMF $\times$ 5 time windows $\times$ 3 features), in setups 3 and 5 Random Forest selected 888 of 2025 features (27 channels $\times$ 5 IMF $\times$ 5 time windows $\times$ 3 features), finally in setup6, 2106 of 5103 features were selected (27 channels $\times$ 7 frequency bands $\times$ 9 time windows $\times$ 3 features) also by Random Forest, but using frequency bands instead of IMF. We can observe a pattern of equivalence between the compared classifiers. None of them explicitly excel in any of the metrics.

**Table 2:** Result of the mean and standard deviation of the metrics of the experiments.

	MLP		SRC		SRMLP	
Exp. ID	Acc	Kappa	Acc	Kappa	Acc	Kappa
setup1	33.37 ± 4.36	0.00 ± 0.07	34.96 ± 4.07	0.02 ± 0.06	33.70 ± 5.08	0.01 ± 0.08
setup2	32.82 ± 4.41	−0.01 ± 0.07	34.13 ± 5.21	0.01 ± 0.08	34.02 ± 5.40	0.01 ± 0.08
setup3	32.58 ± 5.17	−0.01 ± 0.08	33.05 ± 6.60	−0.00 ± 0.10	35.50 ± 5.15	0.03 ± 0.08
setup4	34.01 ± 1.87	0.01 ± 0.03	35.08 ± 2.47	0.03 ± 0.04	34.88 ± 2.26	0.02 ± 0.03
setup5	35.10 ± 4.69	0.03 ± 0.07	34.49 ± 4.89	0.02 ± 0.07	33.33 ± 3.60	0.00 ± 0.05
setup6	32.06 ± 5.51	−0.02 ± 0.08	32.60 ± 4.41	−0.01 ± 0.07	33.67 ± 5.14	0.00 ± 0.08

For each setup, 3$\times$ cross-validation (10-fold) were performed, totaling 30 scores per metric.

**Figure 7:**
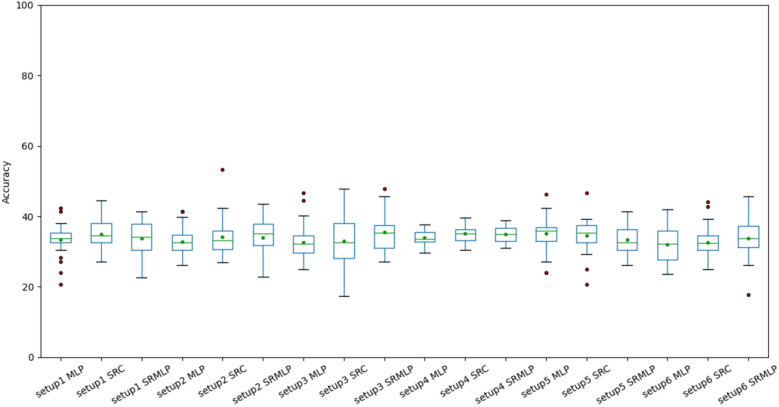
Accuracies of the MLP, SRC and hybrid (SRMLP) classifiers, grouped by setup. We observed a similarity in the mean and median across all approaches, around 34%. We also look at similar distributions, making it difficult to determine from these results whether one setting is superior to another.

**Figure 8:**
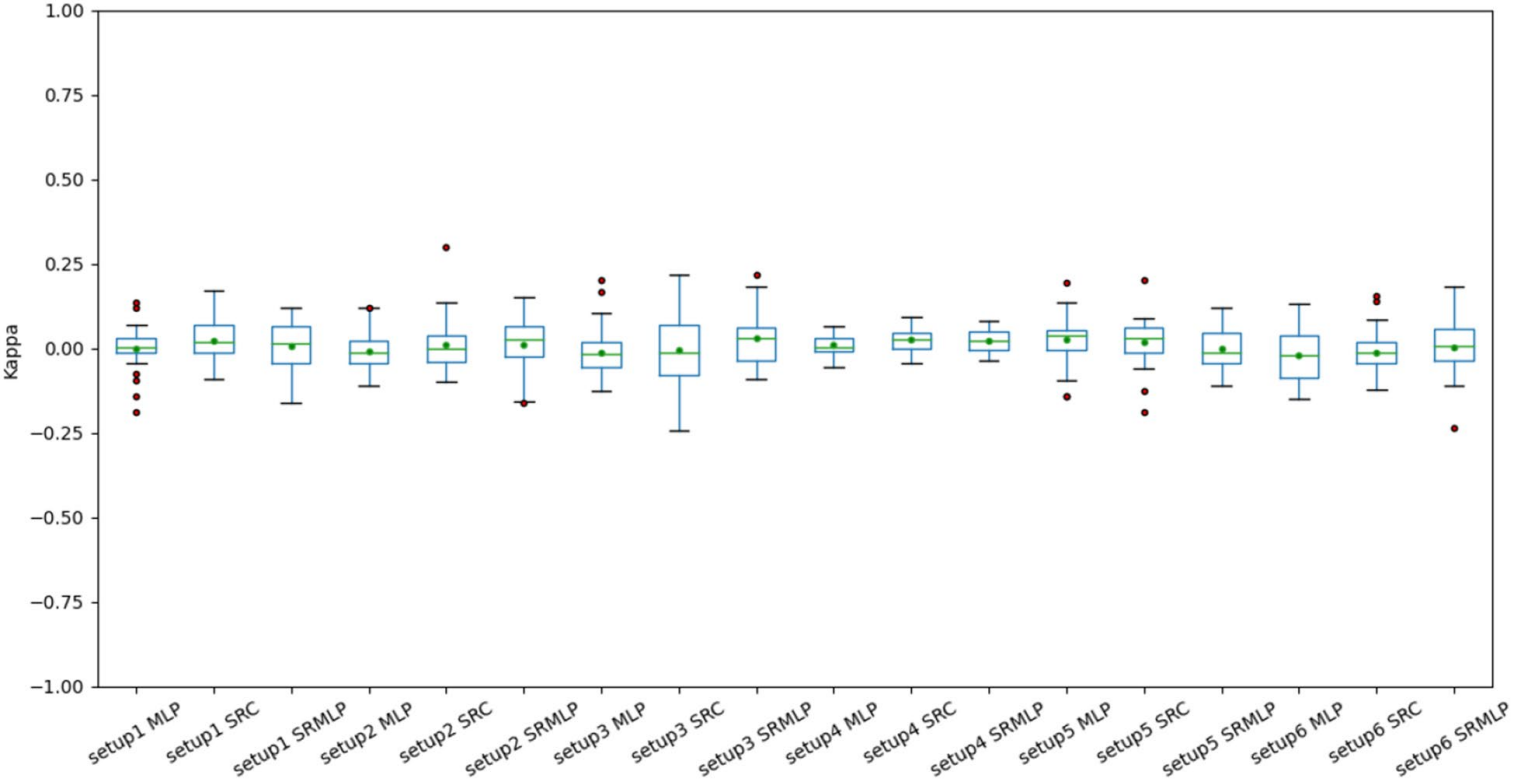
Kappa coefficients of MLP, SRC and hybrid (SRMLP) classifiers, grouped by setup. We observe a behavior similar to Fig. 7 with coefficient oscillating throughout the value 0.

In Fig. 7 we can observe the distribution of accuracies presented in Table 2. Each boxplot has 30 accuracy values, derived from cross validation. Some of the configurations have high interquartile range, having high variance in the results. The smallest variances are observed in the classification of segments (setup 4), since there is a greater volume of data in this base, reducing the unpredictability of the classification. The same pattern can be seen in Fig. 8. However, the results are still far from being of practical use.

The low performance of the metrics suggests that none of the approaches can generalize from the base adopted in this work. However, the averages and deviation obtained are very similar and the high variance suggests a tendency to underfitting. Our hypothesis is that the amount of sample in the base is not enough to discriminate well between classes due to the high sample variance. In this sense, we decided to synthetically increase the dataset, seeking to reduce the variance of the database by using the SMOTE method^83^, which try to preserve the statistical behavior of the data. The Tables 3 and 4 present the results obtained by increasing the base by 30% and doubling the base size, respectively. Data augmentation was not performed in setup 2, due to the high computational complexity of the PSO for large volumes of data. SMOTE was not applied in setup 5 either, as it already performs another data augmentation method, resampling.

**Table 3:** Result of the mean and standard deviation of the metrics of the experiments increasing the base by 30%.

	MLP		SRC		SRMLP	
Exp. ID	Acc	Kappa	Acc	Kappa	Acc	Kappa
setup1	48.62 ± 4.01	0.23 ± 0.06	49.35 ± 4.79	0.24 ± 0.07	**49.43 ± 4.16**	0.24 ± 0.06
setup3	59.89 ± 4.37	0.40 ± 0.07	66.72* ± 3.77	**0.50 ± 0.06**	66.02 ± 4.55	0.49 ± 0.07
setup4	45.12 ± 2.49	0.18 ± 0.04	63.21* ± 2.14	**0.45 ± 0.03**	61.41 ± 2.07	0.42 ± 0.03
setup6	62.83 ± 5.75	0.44 ± 0.09	50.63 ± 3.69	0.26 ± 0.06	66.01* ± 4.06	**0.49 ± 0.06**
Avg	54.11	0.31	57.48	0.36	**60.72**	**0.41**
Vs. MLP (%)	–	–	+6.23	+16.13	**+12.22**	**+32.26**

The (*) mark the statistical difference for *p* value < 0.05 between the MLP approach and the best approach that used sparse representation. Setup 2 is missing, due to the computational complexity of the PSO for large volumes of data. Setup 5 is also missing, as it consists of another data augmentation method, resampling.

Significant values are in bold.

We observe in Table 3 that SRMLP achieved the best average accuracy ($60.72\%$) and a higher percentage increase over MLP ($12.22\%$). The same can be seen with regard to Kappa. SRMLP gets the highest value (0.41) and the highest percentage increase compared to MLP ($32.26\%$). The Mann Whitney test was performed to look for statistical difference for *p* value < 0.05 between the MLP approach and the best approach that used sparse representation and the following *p* values were obtained: setup1 = 0.40, setup3 = 4.82E−07, setup4 = 2.98E−11, setup6 = 8.41E−10 . When we increased the base by 100% (Table 4) we noticed the same evolution of the SRC and SRMLP in relation to the average accuracy, obtaining the highest value ($82.51\%$), meaning an increase of $14\%$ in relation to MLP. Kappa also represents a high percentage increase ($25.42\%$). Finally, SRC outperformed all other methods in setups 3 and 4, second only to the hybrid method (SRMLP) in setup1 (heuristic approach) and setup6 (with time-frequency optimization). The Mann Whitney test was performed and the following *p* values were obtained: setup1 = 0.97, setup3 = 9.78E−11, setup4 = 3.00E−11, setup6 = 2.88E−11.

**Table 4:** Result of the mean and standard deviation of the experiments metrics increasing the base by 100%.

	MLP		SRC		SRMLP	
Exp. ID	Acc	Kappa	Acc	Kappa	Acc	Kappa
setup1	62.40 ± 3.30	0.44 ± 0.05	62.85 ± 3.77	0.44 ± 0.06	**64.52 ± 3.22**	**0.47 ± 0.05**
setup3	84.37 ± 3.23	0.77 ± 0.05	91.43* ± 1.78	**0.87 ± 0.03**	90.44 ± 2.25	0.86 ± 0.03
setup4	55.94 ± 2.37	0.34 ± 0.04	87.06* ± 1.18	**0.81 ± 0.02**	85.31 ± 1.17	0.78 ± 0.02
setup6	86.82 ± 1.98	0.80 ± 0.03	62.48 ± 1.55	0.44 ± 0.02	89.79* ± 2.36	**0.85 ± 0.04**
Avg	72.38	0.59	75.95	0.64	**82.51**	**0.74**
Vs. MLP (%)	–	–	+4.93	+8.47	**+14.00**	**+25.42**

The (*) mark the statistical difference for *p* value < 0.05 between the MLP approach and the best approach that used sparse representation. Setup 2 is missing, due to the computational complexity of the PSO for large volumes of data. Setup 5 is also missing, as it consists of another data augmentation method, resampling.

Significant values are in bold.

**Figure 9:**
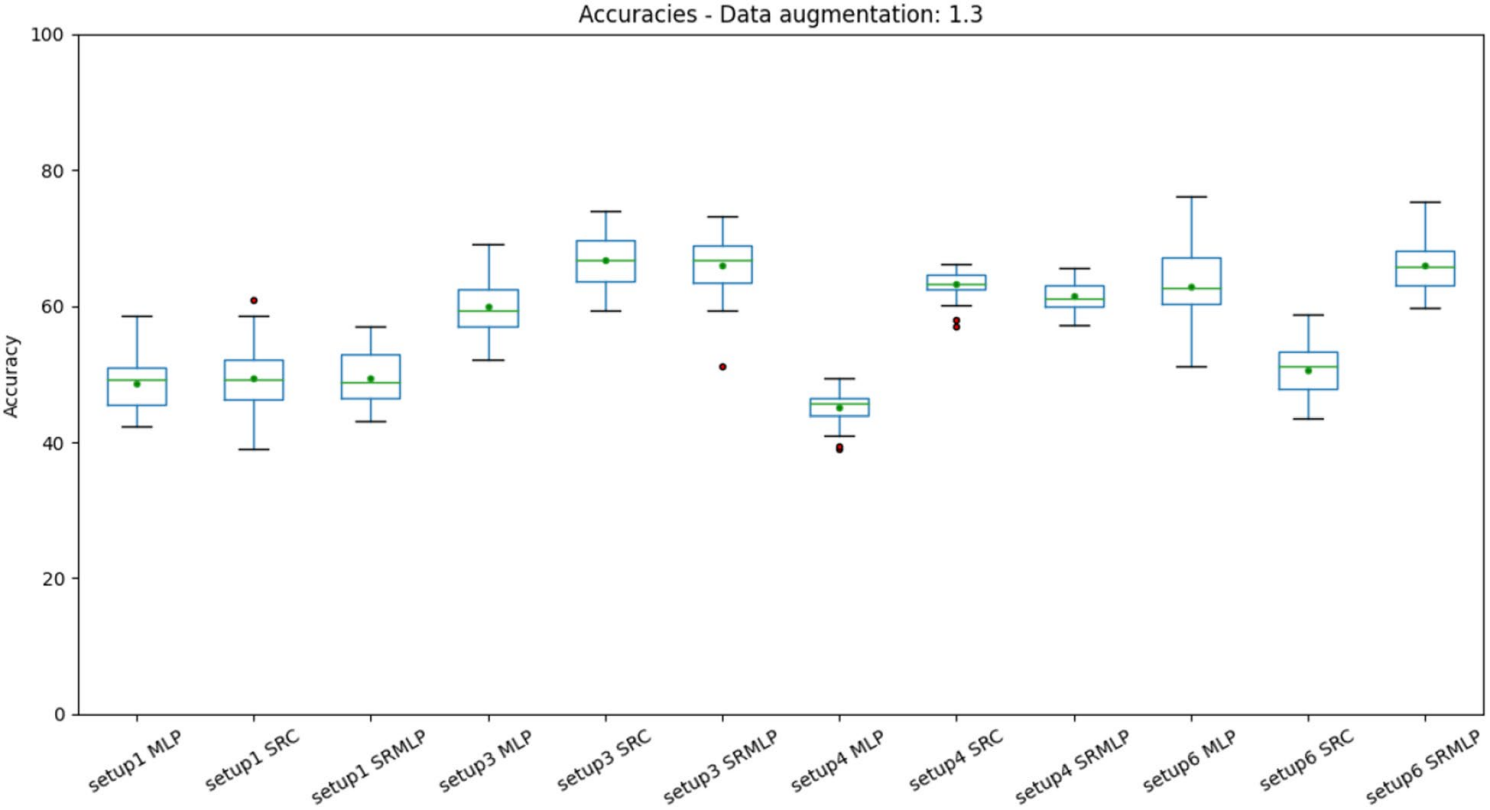
Distribution of accuracies with a 30% increase in base size, grouped by setup. Setups 3 (IMFs) and 6 (Frenquency band) appear a little above the rest. It is also possible to observe a small difference between the pure MLP approach and the approaches that use sparse representation. This is most evident in setup 4, which has a larger volume of data by sorting segments (1s) rather than trials (8s).

**Figure 10:**
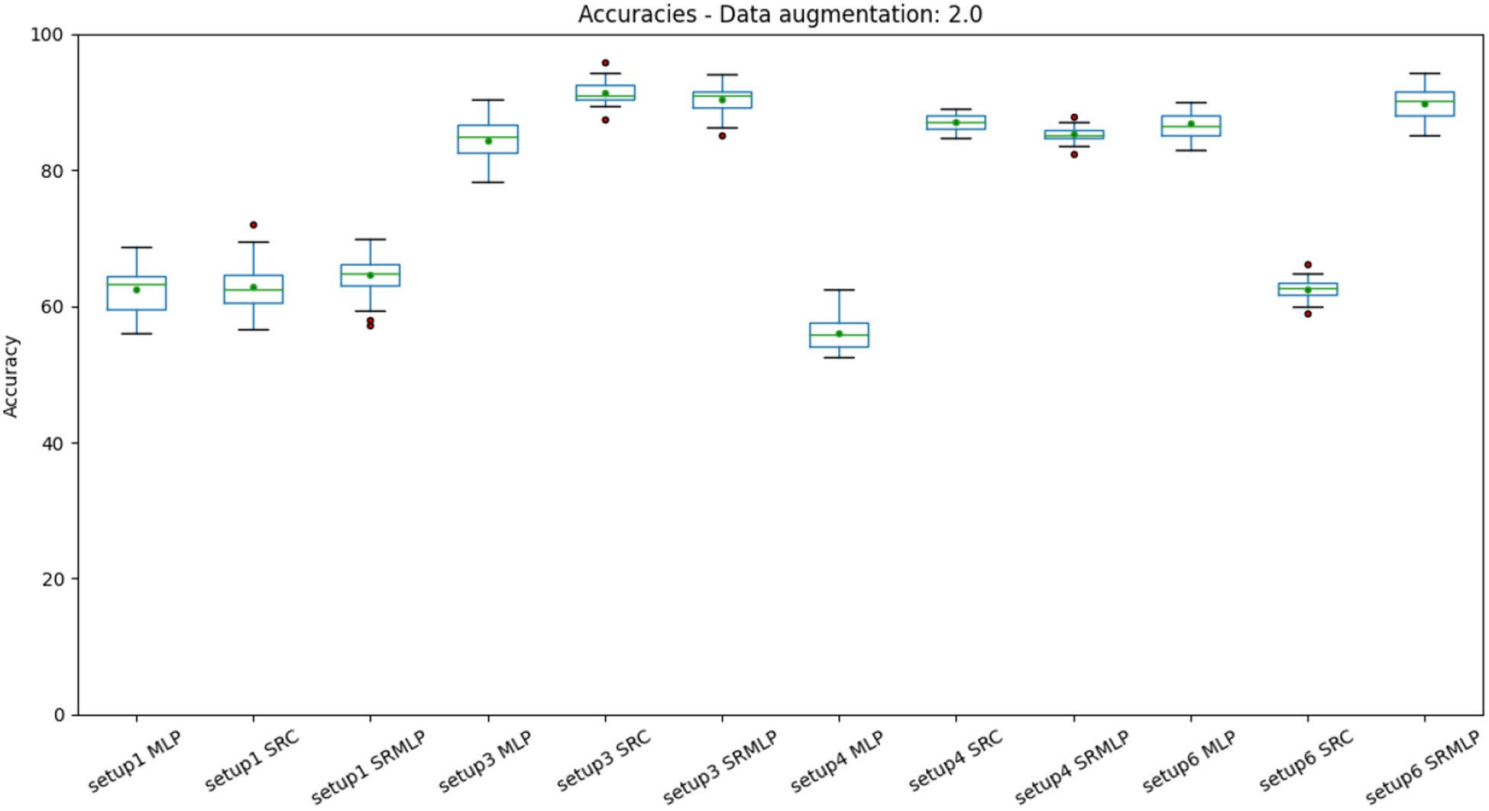
Distribution of accuracies with a 100% increase in base size, grouped by setup. The differences are most notable. Setup 1 (heuristic approach) is left behind, while the other methods achieve good values. It is also possible to notice the decrease in the accuracy variance in relation to the previous figures. The SRC and SRMLP methods outperform MLP but are very similar to each other.

**Figure 11:**
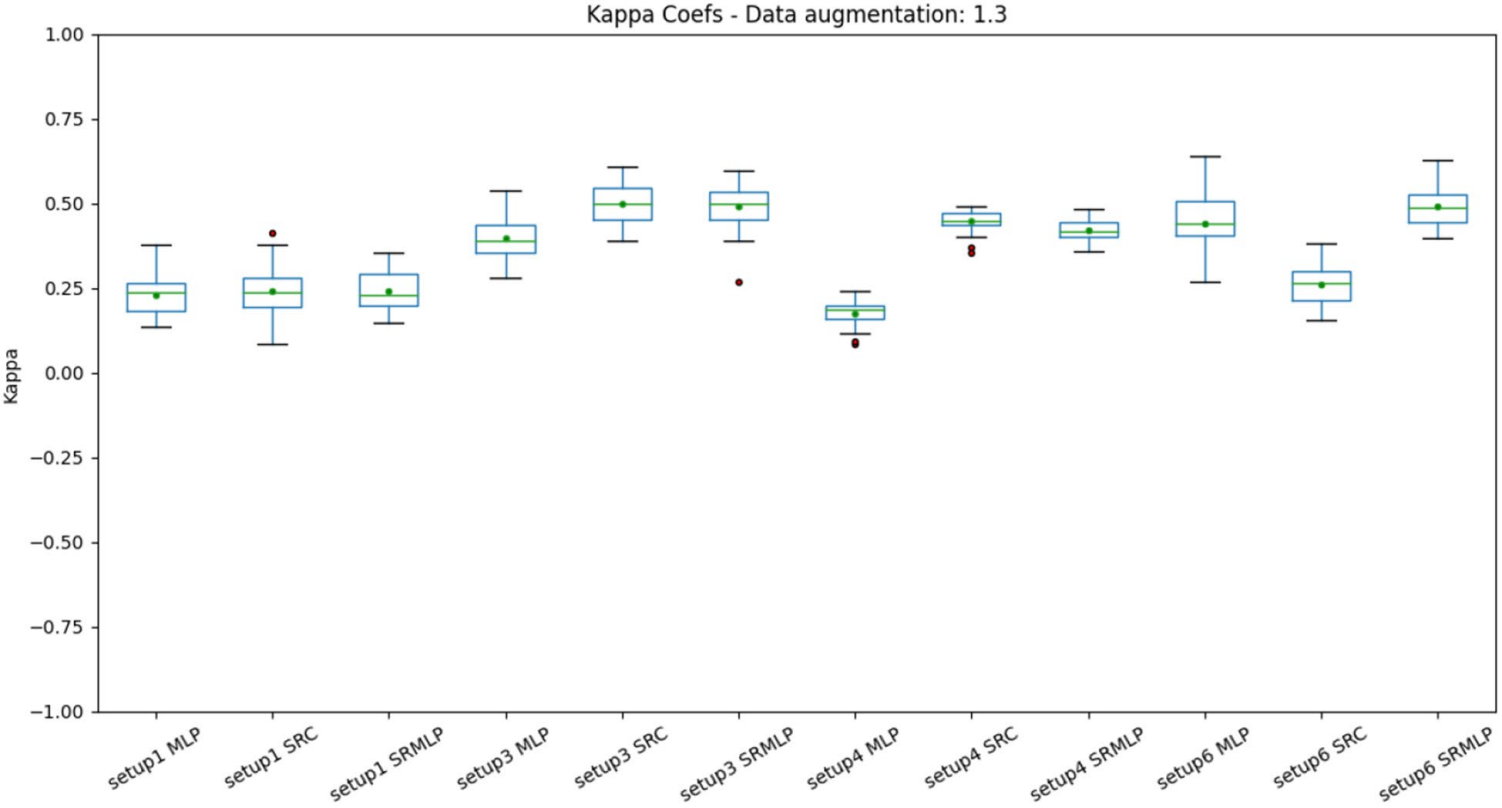
Distribution of Kappa coefficients with a 30% increase in base size, grouped by setup. The results show the same patterns observed in accuracy for the same base size, signaling an improvement in setups 3 and 6 and a difference between pure MLP and sparse representations.

**Figure 12:**
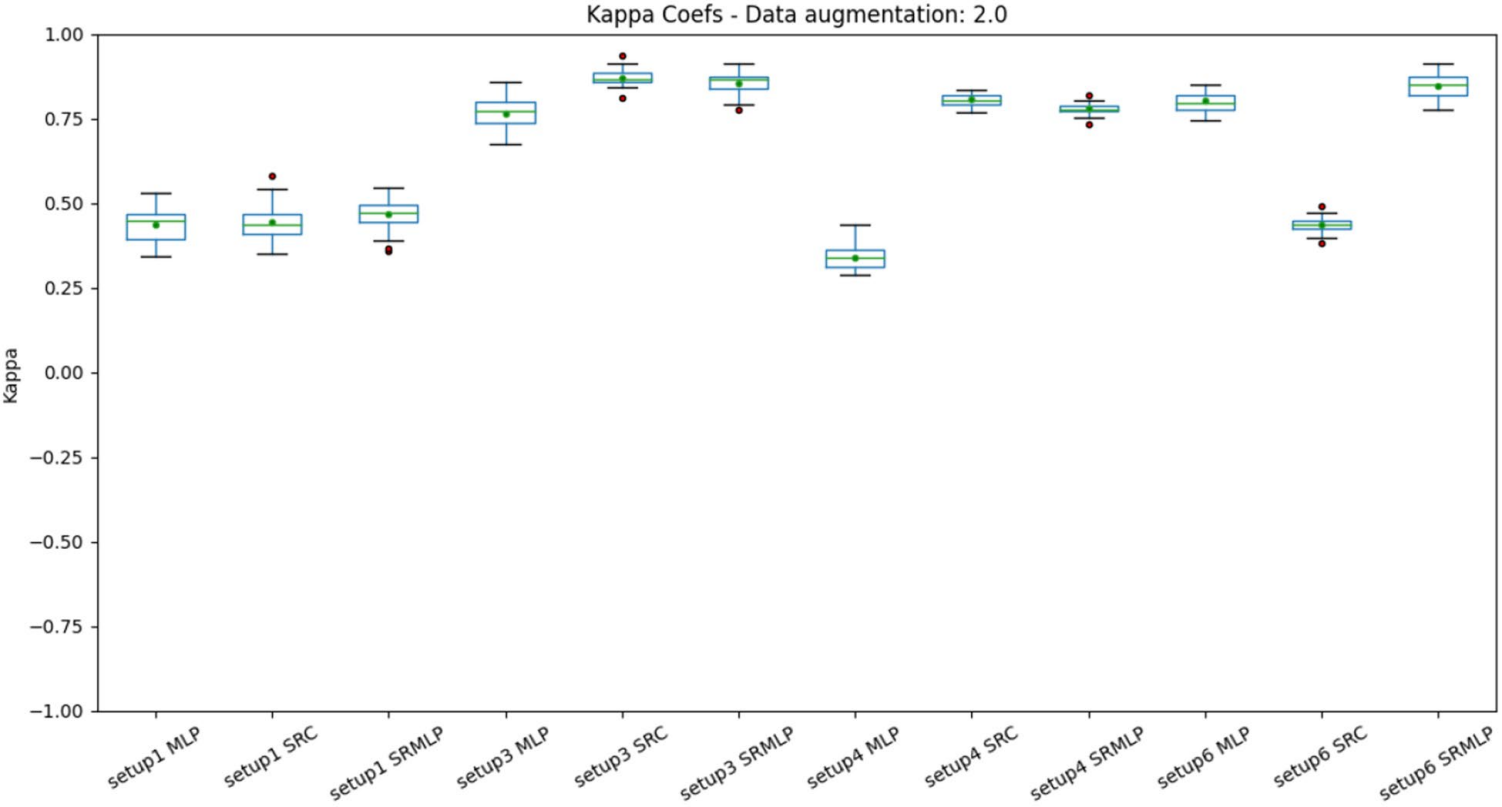
Distribution of Kappa Coefficients with 100% increase in base size, grouped by setup. The results show the same patterns observed in accuracy for the same base size (Fig. 10).

When we look at the boxplots of the accuracies (Figs. 9, 10), we see a proportional increase in intra-setup approaches. It means that all approaches react positively to the increase in the training set and SMOTE does not seem to privilege a specific method. Setups 3 and 6 appear a little above the others and have in common the selection of features through Random Forest. We also noticed that the more we increased the base size, the smaller the variance of the results, the better the precision of the methods. There is a similarity in the distribution of results between the methods that use some sparse representation. Both have a certain statistical equivalence. On the other hand, the difference between an MLP and the other approaches is remarkable. The distributions of the Kappa coefficients, when we increase the base (Figs. 11, 12), behave similarly to the accuracy distributions. Setups 3 and 6 perform better than the others in almost all classifiers. The variance decreases as the volume of data increases.

**Figure 13:**
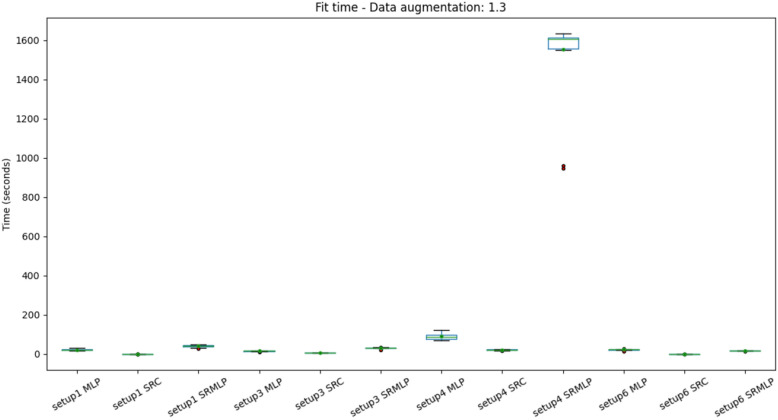
Distribution of training time with a 30% increase in base size, grouped by setup. The classification of segments using SRMLP (setup 4) has a very high cost compared to others. In general the variance is very small, as the size and features of the base are similar between the cross-validation folds.

**Figure 14:**
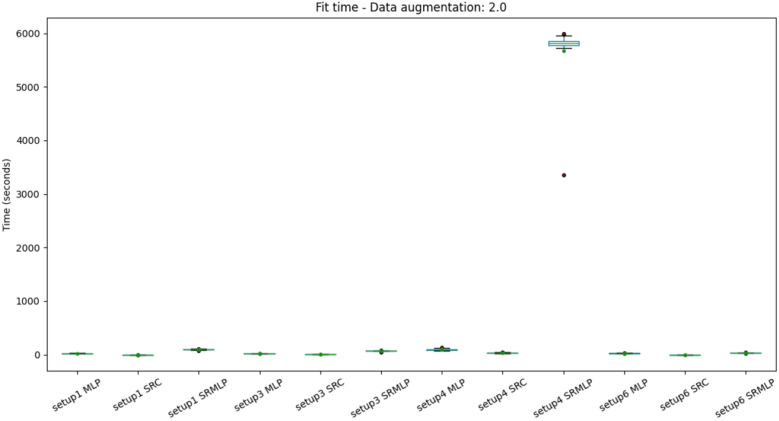
Distribution of training time with 100% increase in base size, grouped by setup. Again, the discrepancy between the 4 SRMLP setup and the other configurations is observed.

We can also observe the results from the perspective of computational complexity. Since the volume of data needs to grow to achieve good results, the more expensive it will be to process. Figures 13 and 14 show the distribution of the average training time for each model. The experiments were performed on a common computer (CPU with 2 cores at 2.50 GHz and 4 threads). In general, training times are similar and should have little impact on the choice of algorithm in practice. Except when it comes to the hybrid approach in setup 4 which is highly at variance with the others. If we look at the magnitude of the training time, we note that this setup must be chosen with care, as it takes more than 20 minutes (1200 s) to train a model. It is also important to note that the same setup SRC approach has equivalent performance with a much lower computational cost.

**Figure 15:**
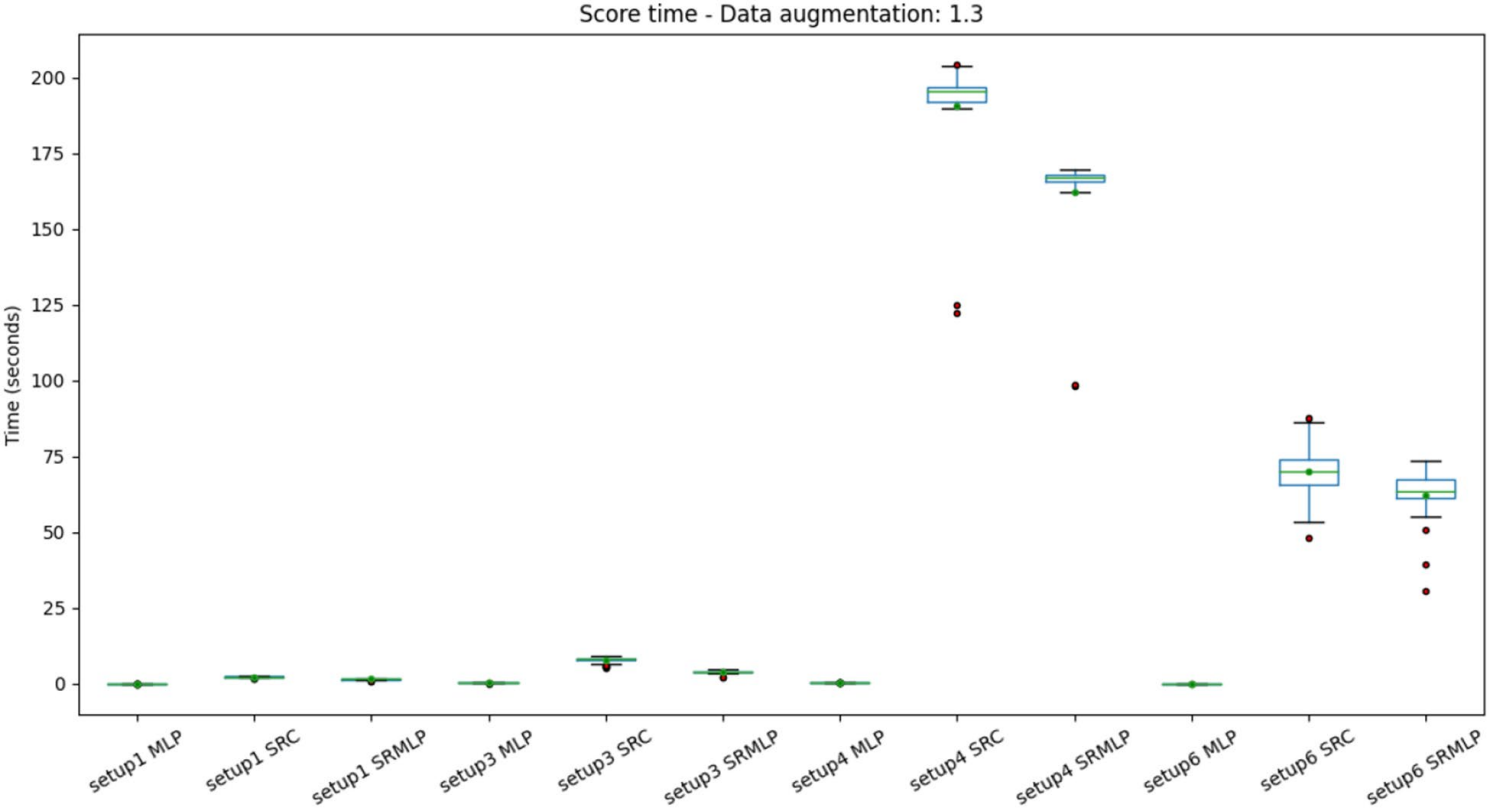
Distribution of prediction time (test in cross-validation) with a 30% increase in base size, grouped by setup. We observed a high time in setup 4 approaches that use sparse representations. However setup 4 consists of segment classification (1s) rather than trials (8s), so it is the base with the most data in your test set.

**Figure 16:**
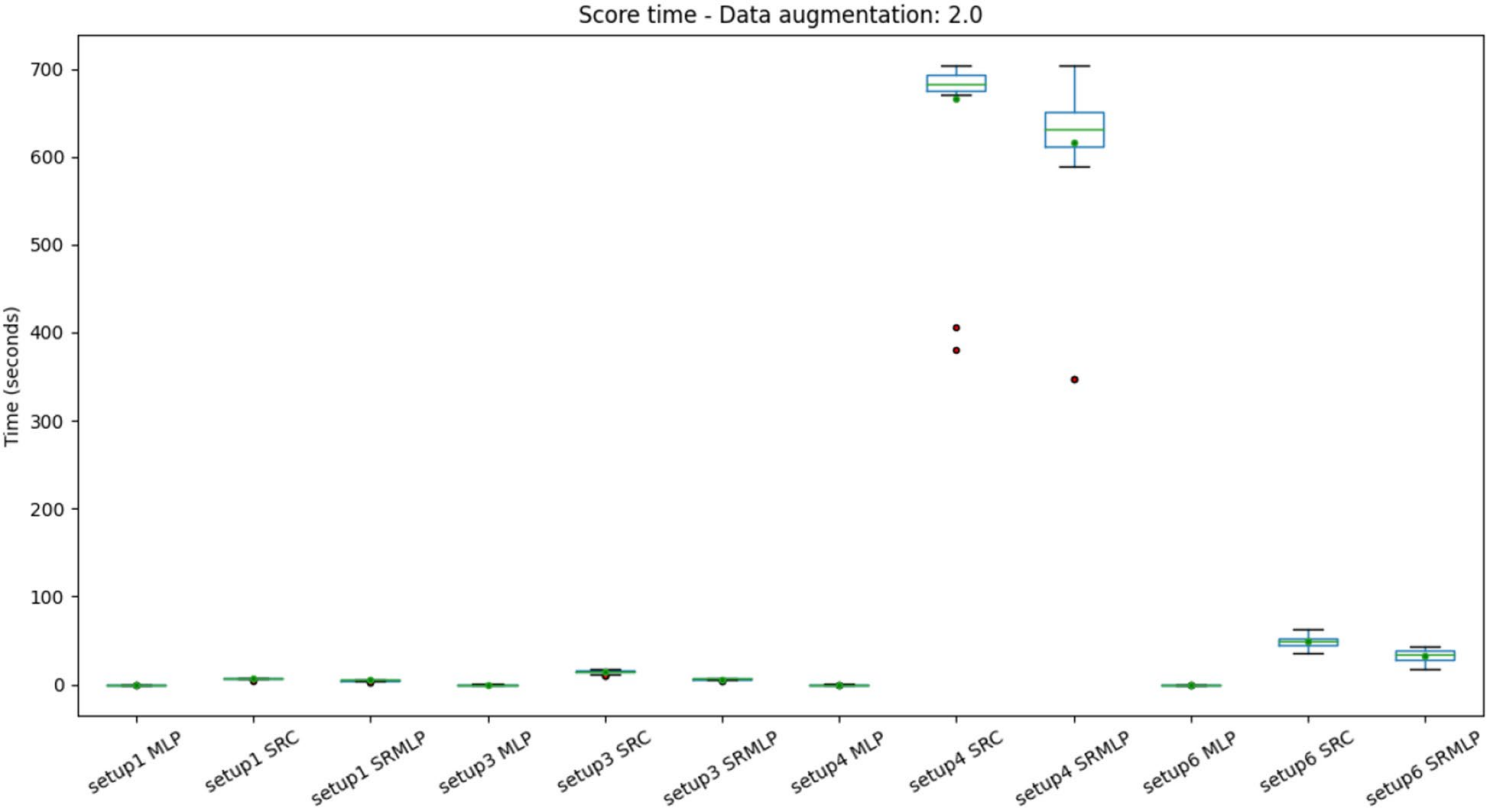
Distribution of the prediction time (test in cross-validation) with the increase of 100% of the base size, grouped by setup. Shows the same pattern as Fig. 15.

Finally, we can assess the computational complexity of classification time. The Figs. 15 and 16 present the distribution of the average classification time of 1 partition of the cross validation. That is, it is not about the time to classify a sample, but the test set. In this sense, we noticed a trend of cost increase also for the SRC in the classification of segments (setup4). However, in practice, segment classification should be intended for online applications, whose prediction is given one sample at a time. This can mitigate the effect of computational cost.

### Database 2: BCI competition IV 2b

To improve classification accuracy we generate artificial training data based on real training samples. The data augmentation method is very similar to the one presented by^33^ which shows the effectiveness of the method. Trials were segmented into three crops and randomly recombined between trials of the same class and subject, generating new samples. Then the decomposition method is applied (EMD or filtering by frequency bands, according to the experiment setup) and the components are also randomly recombined to generate new samples. This method was applied to triple the size of the training base.

**Figure 17:**
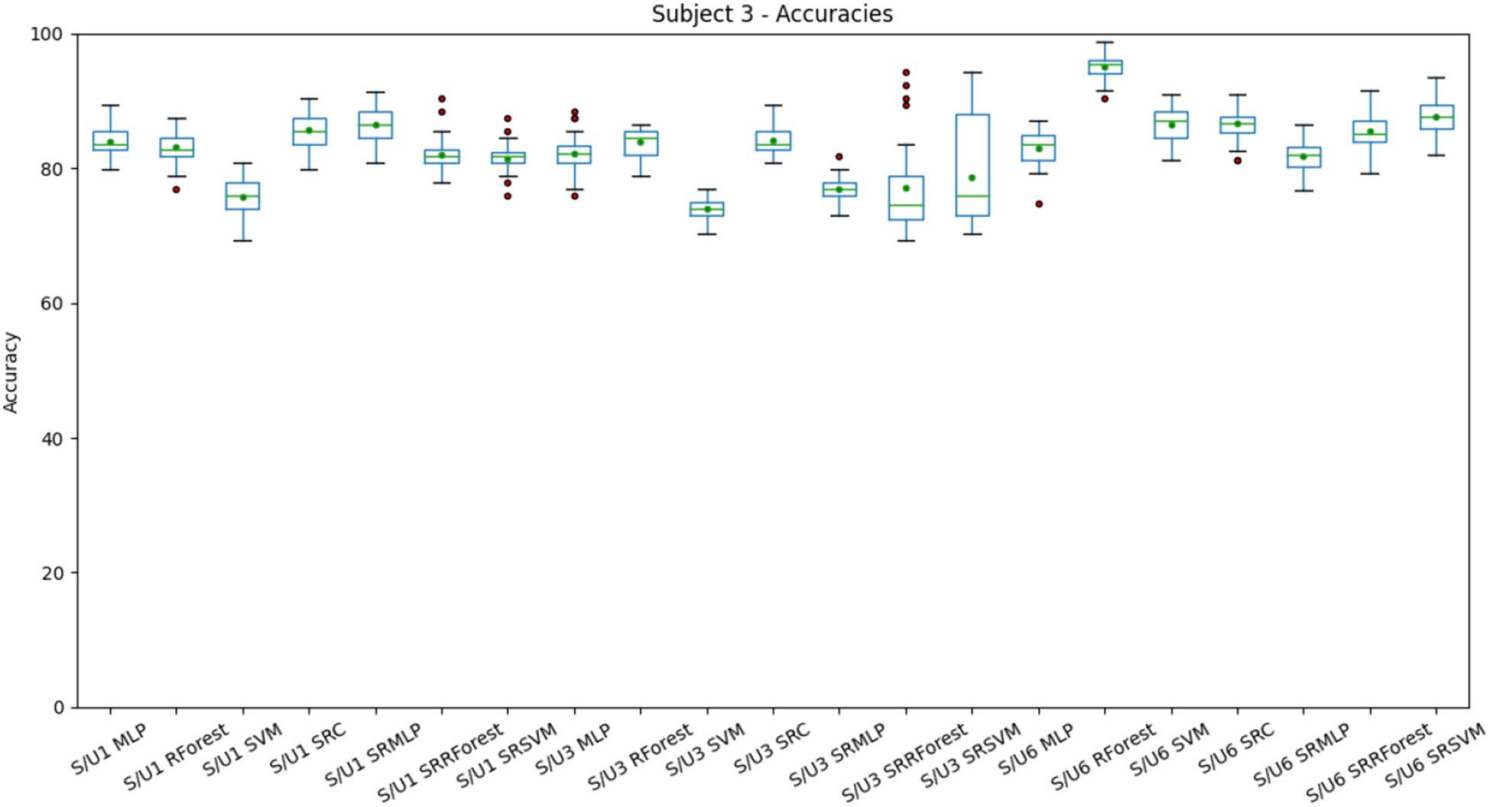
Distribution of subject 3 accuracies, grouped by setup. The differences are more notable. The equivalence between the decomposition methods continues to be observed. We observed a good performance of classifiers that use sparse representation, but doesn’t outperform other approaches. A different result from the first dataset.

In addition to the classifiers used in the experiments with the previous base (MLP, SRC and SR+MLP), Ramdom Forest (RForest) and SVM were also tested. These algorithms were also used in the classification of the space representation (SRR-Forest and SRSVM). Table 5 summarizes the results obtained for each of the 9 subjects in the base (S1–S9) in 3 setups. Setup1 describes the condition in which the experiment does not perform any selection of features, since the selection of C3, Cz and C4 is not necessary, since the base already consists of using the same channels. Setups 3 (with EMD) and 6 (with frequency bands) are presented here to allow again the comparative analysis between these techniques. In setup3, 48 out of 135 features were selected (3 channels $\times$ 5 IMF $\times$ 3 time windows $\times$ 3 features) and in setup6, 114 out of 441 features were selected (3 channels $\times$ 7 frequency bands $\times$ 7 time windows $\times$ 3 features). The Mann Whitney test was performed to look for statistical difference for *p* value < 0.05 between the best approach with a conventional classifier (Random Forest) and the best approach that used sparse representation. Following *p* values were obtained: S1setup1 = 1.93E−09, S1setup3 = 0.56, S1setup6 = 9.07E−08, S2setup1 = 0.84, S2setup3 = 1.83E−10, S2setup6 = 6.80E−11, S3setup1 = 2.70E−06, S3setup3 = 0.007, S3setup6 = 1.64E−10, S4setup1 = 2.66E−11, S4setup3 = 1.29E−11, S4setup6 = 1.14E−06, S5setup1 = 0.004, S5setup3 = 5.43E−11, S5setup6 = 2.04E−05, S6setup1 = 0,80, S6setup3 = 1.39E−06, S6setup6 = 7.86E−07, S7setup1 = 0.0004, S7setup3 = 0.0002, S7setup6 = 3.23E−06, S8setup1 = 0.04, S8setup3 = 2.65E−11, S8setup6 = 5.77E−08, S9setup1 = 3.13E−05, S9setup3 = 0,002, S9setup6 = 3.87E−07. Finally, Fig. 17 presents the distribution of accuracies for subject 3. The same behavior can be observed in the other subjects and can be deduced from the standard deviation values of Table 5.

**Table 5:** BCI CIV 2b—Result of the mean and standard deviation of the accuracies of the experiments.

Exp. ID	S1	S2	S3	S4	S5	S6	S7	S8	S9	AVG
setup1 MLP	89.48 ± 1.91	89.12 ± 1.82	84.01 ± 2.4	96.19 ± 1.05	90.67 ± 1.9	87.8 ± 1.96	88.32 ± 2.0	83.75 ± 2.05	81.5 ± 1.93	87.87 ± 4.13
setup1 R.Forest	91.22 ± 1.65*	86.99 ± 1.79	83.08 ± 2.38	98.94 ± 0.52*	92.38 ± 1.35*	87.4 ± 1.93	89.86 ± 1.91*	88.03 ± 1.26*	83.69* ± 2.44	89.07 ± 4.55
setup1 SVM	89.11 ± 1.54	81.55 ± 1.48	75.71 ± 2.84	98.3 ± 0.76	86.03 ± 1.97	84.62 ± 1.83	88.75 ± 1.52	83.37 ± 2.14	79.43 ± 1.64	85.21 ± 6.15
setup1 SRC	88.17 ± 2.23	87.08 ± 1.77	85.64 ± 2.43	88.56 ± 1.97	86.13 ± 2.36	85.69 ± 2.62	91.03 ± 1.87	78.18 ± 2.38	72.46 ± 2.07	84.77 ± 5.47
setup1 SRMLP	86.7 ± 2.1	86.81 ± 1.84	86.51 ± 2.39*	94.75 ± 1.4	91.23 ± 1.57	87.19 ± 2.39	87.81 ± 1.98	86.78 ± 3.04	80.3 ± 3.11	87.56 ± 3.67
setup1 SRR.Forest	88.01 ± 2.0	87.37 ± 1.7	82.08 ± 2.62	95.18 ± 1.39	89.28 ± 2.04	89.51 ± 1.85	88.29 ± 2.05	84.51 ± 1.9	79.67 ± 2.24	87.1 ± 4.29
setup1 SRSVM	87.83 ± 2.63	88.07 ± 1.82	81.47 ± 2.3	94.47 ± 1.41	89.33 ± 1.49	88.9 ± 2.33	88.18 ± 1.74	84.47 ± 2.77	79.49 ± 2.61	86.91 ± 4.23
setup3 MLP	90.31 ± 1.63	88.01 ± 2.09	82.12 ± 2.91	96.36 ± 1.45	90.38 ± 2.08	87.68 ± 2.57	88.03 ± 2.24	80.8 ± 1.28	81.71 ± 2.27	87.27 ± 4.74
setup3 R.Forest	90.18 ± 1.72	88.74 ± 1.65*	84.04 ± 2.32*	98.13 ± 0.54*	92.72 ± 1.49*	87.37 ± 1.2*	88.86 ± 1.64*	87.27 ± 1.31*	79.58 ± 1.41*	88.54 ± 4.9
setup3 SVM	86.18 ± 1.46	81.17 ± 1.56	74.01 ± 1.91	97.07 ± 0.81	85.38 ± 1.46	85.08 ± 1.25	86.64 ± 1.34	82.73 ± 0.91	78.2 ± 1.12	84.05 ± 6.04
setup3 SRC	88.81 ± 1.74	85.26 ± 1.78	84.23 ± 2.21	64.23 ± 1.22	73.77 ± 1.82	72.6 ± 2.56	81.54 ± 2.35	72.12 ± 2.61	64.29 ± 2.41	76.32 ± 8.53
setup3 SRMLP	90.89 ± 2.37	85.12 ± 1.47	76.92 ± 1.6	75.2 ± 2.15	84.77 ± 1.97	82.91 ± 1.84	85.87 ± 2.39	81.14 ± 1.76	73.87 ± 2.61	81.85 ± 5.28
setup3 SRR.Forest	89.42 ± 4.87	84.12 ± 1.82	77.18 ± 6.83	76.1 ± 1.42	85.62 ± 2.33	82.87 ± 3.7	84.9 ± 4.2	81.33 ± 2.25	78.38 ± 3.3	82.21 ± 4.12
setup3 SRSVM	89.69 ± 5.35	83.86 ± 1.59	78.72 ± 7.9	76.12 ± 1.93	86.18 ± 2.29	83.61 ± 3.17	84.5 ± 4.57	80.95 ± 1.9	77.72 ± 2.92	82.37 ± 4.12
setup6 MLP	87.24 ± 2.31	83.21 ± 2.65	83.07 ± 2.61	97.69 ± 1.19	95.42 ± 1.75	89.32 ± 1.93	85.5 ± 2.62	92.14 ± 2.13	89.66 ± 2.21	89.25 ± 4.84
setup6 R.Forest	94.52* ± 2.03	95.63* ± 1.77	95.07* ± 1.91	98.67* ± 0.83	97.14* ± 1.08	94.38* ± 1.87	91.9* ± 2.38	95.31* ± 1.57	94.36* ± 2.16	95.22 ± 1.79
setup6 SVM	89.78 ± 2.15	87.79 ± 2.96	86.51 ± 2.71	97.19 ± 1.31	93.73 ± 1.98	91.87 ± 2.4	88.13 ± 2.49	91.29 ± 2.09	89.16 ± 1.6	90.61 ± 3.14
setup6 SRC	86.16 ± 3.02	85.16 ± 2.81	86.7 ± 2.59	94.63 ± 1.36	91.28 ± 2.2	88.0 ± 2.39	84.31 ± 2.22	86.17 ± 2.7	87.21 ± 2.29	87.74 ± 3.07
setup6 SRMLP	88.93 ± 2.04	82.62 ± 2.23	81.82 ± 2.75	96.79 ± 0.94	96.04 ± 1.38	91.02 ± 2.41	88.06 ± 2.08	94.23 ± 2.16	89.38 ± 2.57	89.88 ± 5.03
setup6 SRR.Forest	88.36 ± 2.85	85.02 ± 2.55	85.48 ± 2.94	97.11 ± 1.55	96.02 ± 1.16	89.7 ± 2.79	88.92 ± 2.99	93.06 ± 1.39	90.38 ± 2.02	90.45 ± 4.0
setup6 SRSVM	90.48 ± 2.34	88.3 ± 2.52	87.69 ± 3.0	97.14 ± 1.12	95.3 ± 1.65	91.34 ± 1.97	88.48 ± 2.38	92.38 ± 1.71	90.74 ± 2.18	91.32 ± 3.02

For each setup, 3$\times$ cross validation (10-fold) was performed, totaling 30 scores. S1–S9 identifies the subjects of the base. The (*) mark the statistical difference for *p* value < 0.05 between the best approach with a conventional classifier (Random Forest) and the best approach that used sparse representation.

Significant values are in bold.

Table 6 considers the most accurate models obtained by the previous experiment and presents the inter-subject average of each setup. The best sparse model (Setup6 SRSVM) achieve an average of $95.43\%$ of average accuracy among the subjects in the base. The best conventional model (Setup6 R.Forest) reaches up to $98.33\%$ accuracy, a very close result between the approaches.

**Table 6:** BCI CIV 2b—Accuracy of the best models of each evaluated configuration.

Exp. ID	MLP	R.Forest	SVM	SRC	SRMLP	SRR.Forest	SRSVM
S1 setup1	92.66	**93.58**	91.74	**93.58**	90.83	92.66	92.66
S1 setup3	93.58	93.58	88.07	91.74	96.33	**99.08**	**99.08**
S1 setup6	92.07	**98.17**	94.51	91.46	93.29	93.29	95.12
S2 setup1	**93.86**	91.23	84.21	91.23	89.47	91.23	91.23
S2 setup3	**92.98**	91.23	84.21	89.47	88.6	88.6	87.72
S2 setup6	88.24	**98.83**	92.35	88.82	87.65	90.06	93.57
S3 setup1	89.42	87.5	80.77	90.38	**91.35**	90.38	87.5
S3 setup3	88.46	86.54	76.92	89.42	81.73	**94.23**	**94.23**
S3 setup6	87.1	**98.71**	90.97	90.97	86.45	91.61	93.55
S4 setup1	98.58	**99.29**	**99.29**	92.91	98.58	97.87	97.87
S4 setup3	**98.58**	**98.58**	**98.58**	67.38	82.98	80.14	82.98
S4 setup6	**100.0**	**100.0**	99.53	97.16	98.58	**100.0**	99.05
S5 setup1	**94.62**	**94.62**	90.77	90.0	93.85	**94.62**	91.54
S5 setup3	94.62	**95.38**	87.69	78.46	88.46	89.23	91.54
S5 setup6	98.97	**99.48**	97.93	94.85	98.45	97.94	97.93
S6 setup1	91.74	91.74	88.07	91.74	91.74	91.74	**93.58**
S6 setup3	**91.74**	88.99	87.16	79.82	87.16	88.07	88.07
S6 setup6	93.17	**96.89**	96.27	93.79	95.65	95.65	95.65
S7 setup1	92.31	93.16	91.45	**94.87**	91.45	92.31	91.45
S7 setup3	92.31	92.31	89.74	88.03	91.45	92.31	**93.16**
S7 setup6	90.23	**96.55**	92.53	89.6	93.64	94.25	93.06
S8 setup1	86.36	89.77	87.5	84.09	**94.32**	88.64	88.64
S8 setup3	82.95	**89.77**	85.23	75.0	85.23	85.23	85.23
S8 setup6	96.99	**98.19**	95.18	91.57	**98.19**	95.78	95.78
S9 setup1	84.68	**88.29**	83.78	76.58	87.39	86.49	84.68
S9 setup3	85.59	82.88	80.18	70.27	80.18	**86.49**	84.68
S9 setup6	93.98	**98.19**	92.77	92.17	93.37	93.98	95.18
AVG setup1	91.58	**92.13**	88.62	89.49	92.11	91.77	91.02
AVG setup3	91.2	**91.03**	86.42	81.07	86.9	89.26	89.63
AVG setup6	93.42	**98.33**	94.67	92.27	93.92	94.73	95.43

Significant values are in bold.

## Discussion

### Database 1

The dataset 1, although voluminous, is still not sufficiently representative for the problem of distinguishing three classes of motor imagery. The performance improvement obtained with SMOTE corroborates this thesis. However, the user cannot be expected to have so much time to obtain a significant volume of data. To have an estimate, each collection has 60 trials of 8 s, which is equivalent to 8 minutes per collection. The original base, without data augmentation, has 21 collections, a total of 168 minutes. However, we only saw a noticeable improvement in the data when we doubled the size of this database (Figs. 10, 12). It is unfeasible for a person to take that long to provide a massive amount of data. In this sense, the improvement of approaches that use the data augmentation mechanism is essential if we want to reduce the user’s reluctance to use BCI. However, the problem is not new. It is possible to observe in the literature the use of data augmentation to increase the size of the training base in EEG signals^33^.

About the performance obtained in each setup. We can observe that in addition to adaptive methods (EMD and SRC), the selection of features greatly impacts the result. The heuristic approach (setup 1) is far from being the most promising, as it grew the least as the base increased (Figs. 9, 10, 11, 12). This signals that the intentional choice of brain regions (C3, C4 and Cz channels) and frequencies (IMF 3 and 4) is not the best choice. It is possible to affirm that motor imagery information may be better distributed in other regions of the motor cortex and in the frequency spectrum. This reinforces the need to use automatic and adaptive mechanisms for feature selection.

Setup 2 uses PSO to select features among all channels and frequencies. It proved to be very costly for feature selection, being deprecated in favor of selection by Random Forest. Setup 3 replaces PSO by selecting Random Forest. It was one of the settings that grew the most as the base increased (Figs. 10, 12). It also presented one of the best performances of the SRC and SRMLP, surpassing the conventional technique. We observe with it the first indication of the effectiveness of sparse representations for classifying motor imagery.

Setup 4 is similar to setup 3, but classifies segments (1s) instead of trials (8s). It is as close to an online rating approach as the user expects real-time feedback (equal to or less than 1 s). It also corroborates the superiority of the SRC and SRMLP in relation to the MLP, presenting a more notable difference. However, when looking at training and testing time, we must consider it carefully, as the larger the training base, the SR approaches become more costly. For this case, it is possible to think about optimizing the training dictionary to reduce its size, since the SR dictionary is the set of all training samples in the base. It is also an option to merge the prediction between the pure (faster) MLP model and an SR model. From the degree of certainty that the MLP classifies its samples, we can resort to an SR model to help classify the more difficult samples, as suggested in^7^. Setup 5 has a similar setup to setup 3, but increasing by 30% the base with resampling. It did not stand out in relation to the other methods (Figs. 7, 8). When it comes to data augmentation, we find it more promising to use SMOTE as it generates new samples close to the group of existing samples.

**Figure 18:**
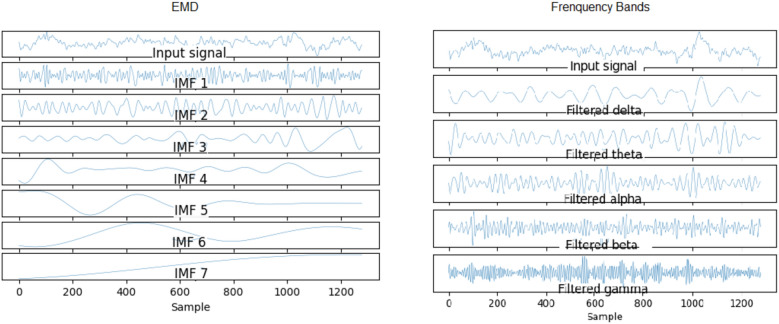
On the left we present the EMD decomposition of an EEG signal. The decomposition for the signal in question generated 7 IMF. On the right we present an EEG signal filtered in frequency bands. It is interesting to note that the 1st IMF has a frequency very similar to the beta filtered signal, while the IMF 2 resembles the signal composition in the alpha and theta bands.

Setup 6 has a configuration similar to setup 3, but instead of using EMD, it decomposes the signal through a bank of frequency filters and overlap the windows in the time domain, thus seeking to detect the best frequency band and window of time for a subject. Similar to setup 3, the SRMLP classifier is superior to the MLP, but the performance of the SRC does not repeat this behavior. For this dataset features based on EMD perform better when associated with the SRC. However, other classifiers have a behavior very similar to that observed in setup 3, improving their performance in the same proportion as the database increases (Figs. 9, 10, 11, 12). In this sense, the use of EMD does not seem to have an impact on most results. It is possible that the similarity of the results is due to the similarity between the IMF and the filtered signal. Figure 18 illustrates a comparison between the models. It is possible to notice the similarity in the frequency of the waves between the two approaches. We can assume this is because brain signals do not have very high frequencies and oscillate in a similar way. EMD has little room to generate IMFs of varying frequencies.

We observed that the classifiers that use sparse representation have equivalent results among themselves, but outperform the conventional MLP model. The SRC and SRMLP reach an average accuracy of $75.95\%$ and $82.51\%$ respectively, while the MLP is $72.38\%$, representing a gain of between $4.93\%$ and $14\%$. Between themselves, the two models that use sparse representation have very similar accuracies, however when time-frequency optimization is used (setup 6) the difference is notable, as the SRC drops in performance. This does not make setup 6 superior to other approaches. It is important to note that the time-frequency optimization does not stand out among the others for this dataset. On the other hand, the use of EMD is also not superior in most cases. However, EMD does not influence negatively, there is room for improvement. Finally, the use of data augmentation proved to be important for obtaining relevant results.

### Database 2: BCI competition IV 2b

The results obtained in the BCI Competition dataset 2b present a different behavior from the first dataset of this study. When comparing the approaches observing the feature processing techniques, it is possible to perceive a superiority of the filtering by frequency bands in relation to the features based on EMD. The average accuracy of the setup 6 models generally exceeds the accuracies of setups 1 and 3 (Table 5). Regarding the classification algorithm, we can also notice that the models that use sparse representation perform slightly inferior to the others in most subjects. Therefore, the model proposed by^7^ in the classification of EMG signals does not seem to be the best when working with EEG data. However, dictionary optimization techniques can help to improve the results, better adapting this approach to the context of motor imagery classification.

When we look at Table 6, the previous observations are even more evident, however this table still allows us to observe which of the tried configurations is the best in the general average. The Random Forest classification of features based on frequency bands (AVG setup6, column 3) is the best configuration among the others, with $98.33\%$ accuracy. It is worth noting that in setup6, the selection of features is also carried out by random forest, which explains the reason for the good performance of this combination.

We can then compare with the cited works that use the same data set. Table 7 presents the performance between the approaches^9^ uses SRC with and without dictionary optimization. However, it only experiences the 3rd collection of each subject, so the training and test data are similar, as they were collected on the same day and time. Therefore, the adaptive factor, where the imagery could vary on different days, cannot be evaluated in these results. The same is in^29^, but it also uses time window and frequency band optimization. The other works, including this one, use the complete base^31^ gives only the average result of all subjects. Our study thus obtains a performance similar to the state of the art. We can feature this performance to the lack of optimization of the sparse dictionary, considering that its optimization proved to be efficient in increasing the accuracy^9^. The synthetic increase in the training base contributed to increase the accuracy of our study. It is interesting to note that it is the same data augmentation technique used by the study that had the best performance, but that uses another^33^ classification method.

**Table 7:** Comparison between the accuracies of different studies for the BCI Competition IV 2b base.

Subject	^9^ SRC	^9^ SRC$+$	^33^	^46^	^29^	^31^	This work (SRC)	This work (SRMLP)
S1	69.43	83.83	80.5	62.8	88.10	–	88.17	86.7
S2	53.00	62.61	70.6	67.1	61.9	–	87.08	86.81
S3	51.24	61.32	85.6	98.7	60.5	–	85.64	86.51
S4	93.15	98.11	94.6	88.4	99.4	–	88.56	94.75
S5	81.23	91.91	98.3	96.3	95.0	–	86.13	91.23
S6	64.15	85.78	86.6	75.3	83.5	–	85.69	87.19
S7	76.96	94.39	89.6	72.2	88.8	–	91.03	87.81
S8	80.03	93.26	95.6	87.8	93.5	–	78.18	86.78
S9	79.05	91.14	87.4	85.3	89.4	–	72.46	80.3
AVG	72.03	86.41	87.6	81.6	84.5	70.0	84.78	87.56

The results of this work refer to the average accuracy of the SRC and SRMLP in setup1.

## Conclusion

In this work, we proposed to evaluate the combination of two adaptive methods to the subject’s context, EMD and SRC, in the classification of three-limb motor imagery. Observing the results, it is possible to affirm that SRC is effective and promising in the detection of motor imagery and can be very useful in BCI applications, generating gains of approximately 14% compared to conventional classifier algorithms. The use of EMD in the extraction of features from EEG signals allowed obtaining results competitive with the state-of-the-art works^12–16,43–45^, with the potential advantage of using an approach that does not fix the separation by frequency band: the IMFs are obtained dynamically, through adaptive filtering that does not fix cutoff frequencies, which is more suitable for non-stationary signals such as EEG signals. Additionally, the evaluation of other datasets can also be useful to better understand the impact of the studied techniques. It is noteworthy here that the problem brought up is multiclass and in the domain of motor imagery it is not a trivial problem, with great variations between databases.

The possibility of combining the methods with other techniques is presented as an option for future work. For example, using Wavelet Transform Decomposition in pre-processing could make the method more noise-resilient^84^. Finally, if we want to decrease the cost and effort to use the BCI and still guarantee good performance we need: (1) to invest efforts in generating new samples to synthetically increase the training base; (2) enhance self-adaptive mechanisms that respond efficiently to the user’s context and serve as an enhancement for joint use with the SRC. It is important to realize that although the proposed method was shown to be comparable with other approaches in data set 2, we cannot rule out the evidence found in the first dataset. If, on the one hand, the BCI Competition dataset 2b contains more subjects (9 subjects vs 1 subject), on the other hand the first dataset has a greater amount of subject data (21 collections vs 5). This only suggests that it is necessary to evaluate approaches on a variety of bases in order to observe prevailing behavior and to evidence in which contexts one technique may be superior to another.

## Data Availability

The first dataset analysed during the current study is available in the Biomedical-Computing-UFPE/Motor-Imagery GitHub repository, at the following URL: https://github.com/Biomedical-Computing-UFPE/Motor-Imagery. The second dataset analysed during this study, BCI Competition IV dataset 2b, is included in this published article and its supplementary information files:^49^.
